# Modular Synthesis of Dendritic Oligo‐Glycerol Cationic Surfactants for Enhanced Antibacterial Efficacy

**DOI:** 10.1002/anie.202425069

**Published:** 2025-04-14

**Authors:** Natalie Hanheiser, Yuhang Jiang, Christian Zoister, Mathias Dimde, Katharina Achazi, Chuanxiong Nie, Yuanyuan Li, Rainer Haag, Abhishek K. Singh

**Affiliations:** ^1^ Institute of Chemistry and Biochemistry Freie Universität Berlin Takustrasse 3 14195 Berlin Germany; ^2^ Forschungszentrum für Elektronenmikroskopie, Core‐Facility BioSupraMol Institute of Chemistry and Biochemistry Freie Universität Berlin Fabeckstraße 36a 14195 Berlin Germany; ^3^ School of Pharmacy Hainan Medical University Haikou Hainan 571199 P.R. China; ^4^ NHC Key Laboratory of Tropical Disease Control Hainan Medical, University Haikou Hainan 571199 P.R. China

**Keywords:** Antibacterial challenges, Cationic surfactants, Click chemistry, Cryo‐TEM, Wound healing

## Abstract

Bacterial infections and antibiotic resistance present an ever‐increasing threat to human health worldwide, and medicine urgently needs new alternatives for the successful treatment of bacterial infections. Cationic surfactants have proven to be effective antibacterial agents due to their ability to disrupt bacterial membranes, inhibit biofilm formation, and combat a broad spectrum of pathogens. We employed a orthogonal click chemistry strategy for the efficient modular synthesis of six novel cationic surfactants. Our results emphasize the strong correlation between the surfactant design and its antibacterial potential. Among these six cationic surfactants we identified a prime candidate, which possessed an impressive antibacterial effect against gram‐positive and gram‐negative bacteria, including drug‐resistant strains. We found that our surfactant can prevent biofilm formation and eradicate already existing biofilms. Cryo‐TEM imaging was used to reveal the membrane‐disrupting properties of the surfactant. In‐vivo wound healing experiments underline the surfactants’ ability to inhibit wound infections. Cationic surfactants often face the challenge of balancing strong antibacterial activity with minimal cytotoxicity. Our strategic design and orthogonal click chemistry approach have enabled precise fine‐tuning of molecular structures to achieve an optimal balance between antibacterial efficacy and biocompatibility, effectively overcoming this critical limitation.

## Graphical abstract

An orthogonal click chemistry approach was used to synthesize a series of cationic surfactants. The results show that an optimal balance between positive charges and hydrophobicity is a crucial factor for the improvement of antibacterial properties. Within our synthesized library, surfactants with a C12 alkyl chain and four positive charges showed remarkable bactericidal activity and antibiofilm effects against drug‐resistant MRSA. In addition, these surfactants significantly promoted wound healing in MRSA‐infected wounds.

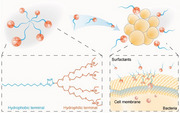

## Introduction

Among pathogenic bacteria, those that have developed resistance to multiple drugs present a special threat to public health.^[^ ^1^, ^2^, ^3^, ^4^ ^]^ The improper and excessive use of antimicrobial agents in agriculture, human, and veterinary medicine promotes the development and spread of resistant bacterial strains through gene transfer, genetic mutations, and biofilm formation. The result has been a steady, ongoing decline in the number of effective treatment options for bacterial infections, highlighting the urgent need for new antimicrobial agents.^[^ ^5^, ^6^ ^]^ One promising class of molecules in this context is cationic surfactants. Surfactants have long been used in biomedical applications,^[^ ^7^, ^8^, ^9^, ^10^, ^11^, ^12^ ^]^ with cationic surfactants containing quaternary ammonium compounds (QACs) particularly noted for their antibacterial and fungicidal activity^[^ ^13^, ^14^, ^15^ ^]^ as well as for their biofilm‐eradicating properties.^[^ ^16^, ^17^, ^18^ ^]^ Because of their positively charged head group, QACs can adhere to the partially negatively charged surface of the bacteria cell via electrostatic interactions. At the same time, their hydrophobic alkyl chains anchor themselves in the bacterial cell wall, leading to its destruction and ultimately to cell death.^[^ ^19^ ^]^ QACs exhibit a broad spectrum of activity against both gram‐positive and gram‐negative bacteria and are therefore widely used as disinfectants and persistence. Nevertheless, antibacterial resistance toward QACs becomes an emerging problem as it was found for methicillin‐resistant *S. aureus* (MRSA).^[^ ^20^, ^21^, ^22^ ^]^ Unfortunately, QACs that are widely used in disinfectants exhibit a strong cytotoxic effect^[^ ^23^, ^24^ ^]^ which can lead to harmful skin irritations.^[^ ^25^, ^26^, ^27^ ^]^ Although problems such as cytotoxicity, growing antibacterial resistance, and biodegradability remain, ongoing research is focused on overcoming these limitations using various chemical approaches to realize QACs’ full potential in medical, pharmaceutical, and industrial applications.^[^ ^23^, ^24^, ^28^, ^29^, ^30^, ^31^, ^32^, ^33^, ^34^, ^35^ ^]^ Their chemical versatility allows fine‐tuning of antibacterial efficacy, selectivity, and biocompatibility. In 2014, Krumm et al. amplified a biocidal quaternary ammonium group to a polymer backbone structure of 2‐methyl‐2‐oxazoline. In addition to that, they introduced a nonbiocidal satellite Group to the polymer. Using this approach they were able to generate a QAC which showed to have a reduced cytotoxic effect and an antibacterial effect against MRSA.^[^ ^36^ ^]^ Another approach was generated by Guo et al., who synthesized a series of cationic amphiphilic dendrons consisting of generation 1 and generation 2 Poly(amidoamine) (PAMAM) with multivalent cationic charges and different hydrophobic alkyl chains via a divergent growth approach. They found out that increasing the chain length of the alkyl chain results in more effective antibacterial activity.^[^ ^37^ ^]^ The synthesis of cationic surfactants involves several challenges, including striking the right balance between antibacterial activity and low cytotoxicity, managing complex multistep reactions, and ensuring the compatibility of functional groups. Scalability and cost are also significant hurdles to large‐scale production, while environmental concerns drive the need for biodegradable and environmentally friendly designs. Overcoming these challenges requires innovative strategies to streamline synthesis and improve the surfactants’ properties for practical applications. The use of oligo‐glycerol based structures for head group design has attracted interest over the last decade. In 2008, Wyszogrodzka et al. were the first to synthesize different generations of polyglycerol‐based dendrons using a convergent growth approach.^[^ ^38^ ^]^ In comparison to PAMAM glycerol is a “green” starting material obtained mainly as a by‐product of the vegetable oil industry.^[^ ^39^, ^40^ ^]^ A key advantage of glycerol and oligo‐glycerol is that they are water‐soluble, biocompatible, and open for postmodification over several hydroxyl groups, making them attractive for biomedical and pharmaceutical applications.^[^ ^41^ ^]^ Especially their lowered cytotoxicity in comparison to other oligomeric backbone structures is compelling.^[^ ^42^, ^43^ ^]^ In the research described here, we have focused on simplifying the surfactants’ synthetic approach while developing a platform for controlled fine‐tuning of chemical functionalities. This orthogonal “click” strategy ensures precise functionalization with high atom economy and enables the integration of tailored hydrophobic and hydrophilic segments.^[^ ^44^, ^45^, ^46^, ^47^ ^]^ The triazole click reaction provided robust linkages in vivo and in vitro for the attachment of hydrophobic alkyl chains, while the thiol‐ene reaction facilitated the introduction of tuneable hydrophilic cationic charges, directly impacting the systems’ cytotoxicity.^[^ ^48^ ^]^ The synergistic application of these orthogonal click reactions provides a streamlined and versatile means of developing well‐defined amphiphilic architectures with improved antibacterial efficacy.^[^ ^49^ ^]^ We synthesized a series of cationic surfactants consisting of low‐generation oligoglycerol (G0‐G2) and a hydrophobic alkyl chain (C12 & C18). By tuning the chain length of the alkyl chain and the number of positively charged tertiary amine groups, we aimed to study the effect of chain length and charge density on the antibacterial and cytotoxic effect of these compounds especially against drug‐resistant bacteria strains. We therefore explored the potential antibacterial effect of our cationic surfactants against *Escherichia coli* and MRSA in vitro and in vivo.

## Results and Discussion

**Scheme 1:**
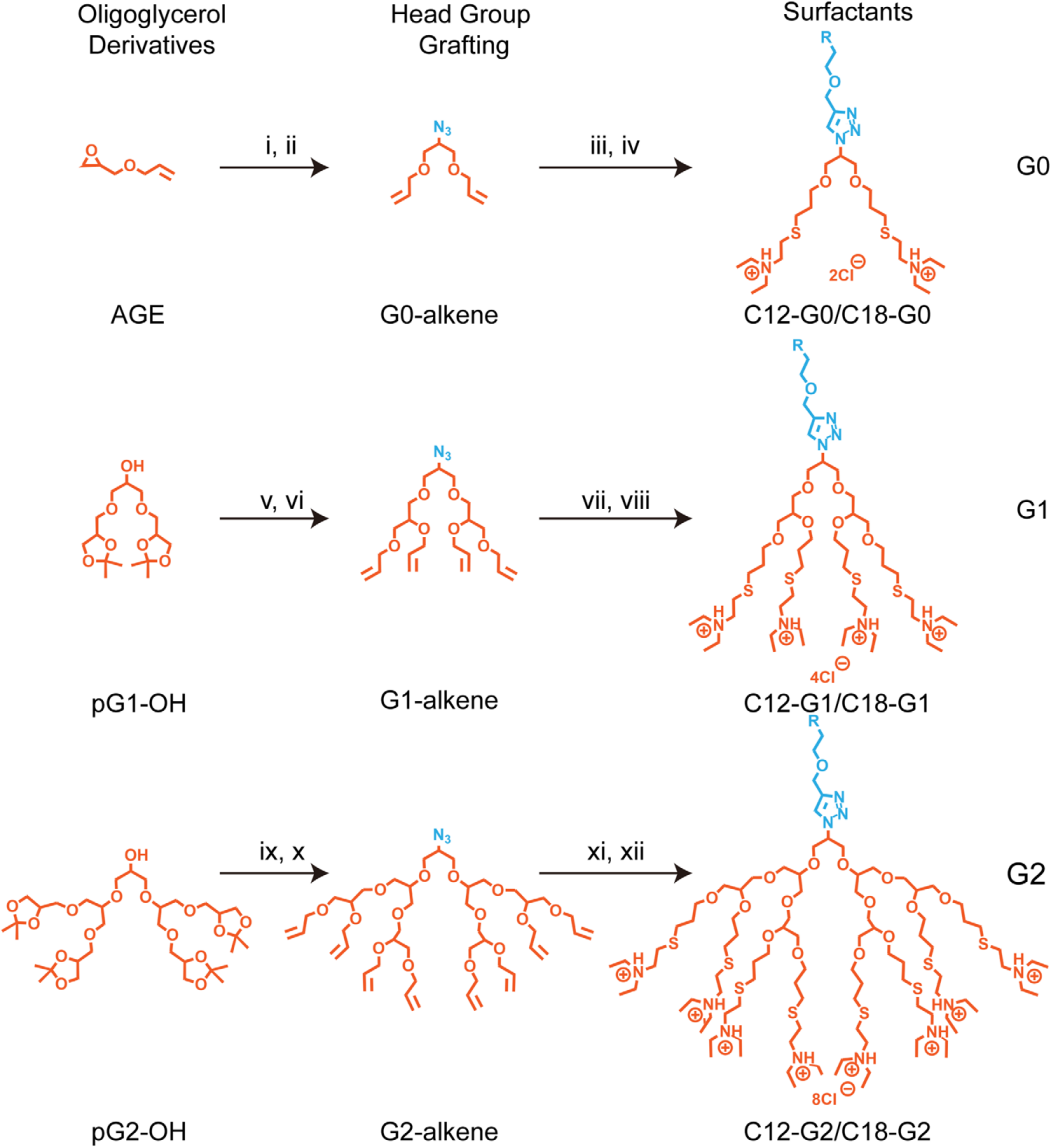
Synthetic procedure for the novel cationic dendritic oligo‐glycerol surfactants with R = C10 or C16. i) Allylalcohol, KOH, TBAB, Toluol, 50 °C, 12 h, and 62%. ii) MsCl, TEA, NaN_3_, DMF, 0‐80 °C, 24 h, and 11%. iii) C12‐alkyne/C18‐alkyne, CuSO_4_, sodium ascorbate, THF/H_2_O, 50 °C, 24 h, 87%/76%. iv) 2‐Diethylaminoethanthiol hydrochloride, DMPA, MeOH, 365 nm, rt, 3 h, and 25%/44%. v) a) MsCl, TEA, NaN_3_, DMF, 0‐80 °C, 24 h, and 81%. b) Dowex‐50WX8, MeOH, H_2_O, 50 °C, 24 h, and 93%. vi) 3‐Brompropene, NaH, DMF, 0‐80 °C, 24 h, and 85%. vii) C12‐alkyne/C18‐alkyne, CuSO_4_, sodium ascorbate, THF/H_2_O, 50 °C, 24 h, and 92%/90%. viii) 2‐Diethylaminoethanethiol hydrochloride, DMPA, MeOH, 365 nm, rt, 3 h, and 90%/82%. ix) a) MsCl, TEA, NaN_3_, DMF, 0‐80 °C, 24 h, and 79%. b) Dowex‐50WX8, MeOH, H_2_O, 50 °C, 24 h, and 89% x) 3‐Brompropene, NaH, DMF, 0‐80 °C, 24 h, and 82% xi) C12‐alkyne/C18‐alkyne, CuSO_4_, sodium ascorbate, THF/H_2_O, 50 °C, 24 h, and 76%/71%, xii) 2‐Dimethylaminoethanethiol hydrochloride, DMPA, MeOH, 365 nm, rt, 3 h, and 74%/68%.

We began our studies by synthesizing a library of six cationic surfactants with varying chain length (C12 and C18) and a varying number of positively charged functional groups (2, 4, and 8). In surfactant chemistry, oligo‐glycerol offers a versatile platform for developing new surfactants for various biomedical applications due to its unique properties, including a high degree of hydrophilicity and the potential to selectively modify any hydroxyl group.^[^ ^43^, ^50^ ^]^ In this study, we synthesized cationic surfactants using different generations of oligo‐glycerol (G0–G2), enabling us to vary the number of multivalent cationic charges on the head group from 2 to 8. To design these cationic surfactants, we employed a orthogonal click chemistry approach: one side to attach the hydrophobic moiety (C12 and C18) using azide‐alkyne click, and the other side to functionalize positive charges using thiol‐ene click. The acetal‐protected G1/G2‐OH were synthesized by following the previously reported literature,^[^ ^38^ ^]^ while G0‐allyl was directly obtained from glycidyl allyl ether (GAE) via epoxide ring opening, using allyl alcohol under basic conditions. To modify the focal point of G0/G1/G2‐OH, we performed mesylation followed by azidation, creating the first clickable site for copper‐catalyzed click chemistry. In the next step, the acetal protection of G1/G2‐N3 was removed under acidic conditions using Dowex 50wx8 cation exchange resin, yielding free hydroxyl groups, which were further allylated using an excess amount of allyl bromide and NaH to complete the conversion of hydroxyl to allyl and provide the second site for thiol‐ene click coupling (Scheme 1).

**Figure 1:**
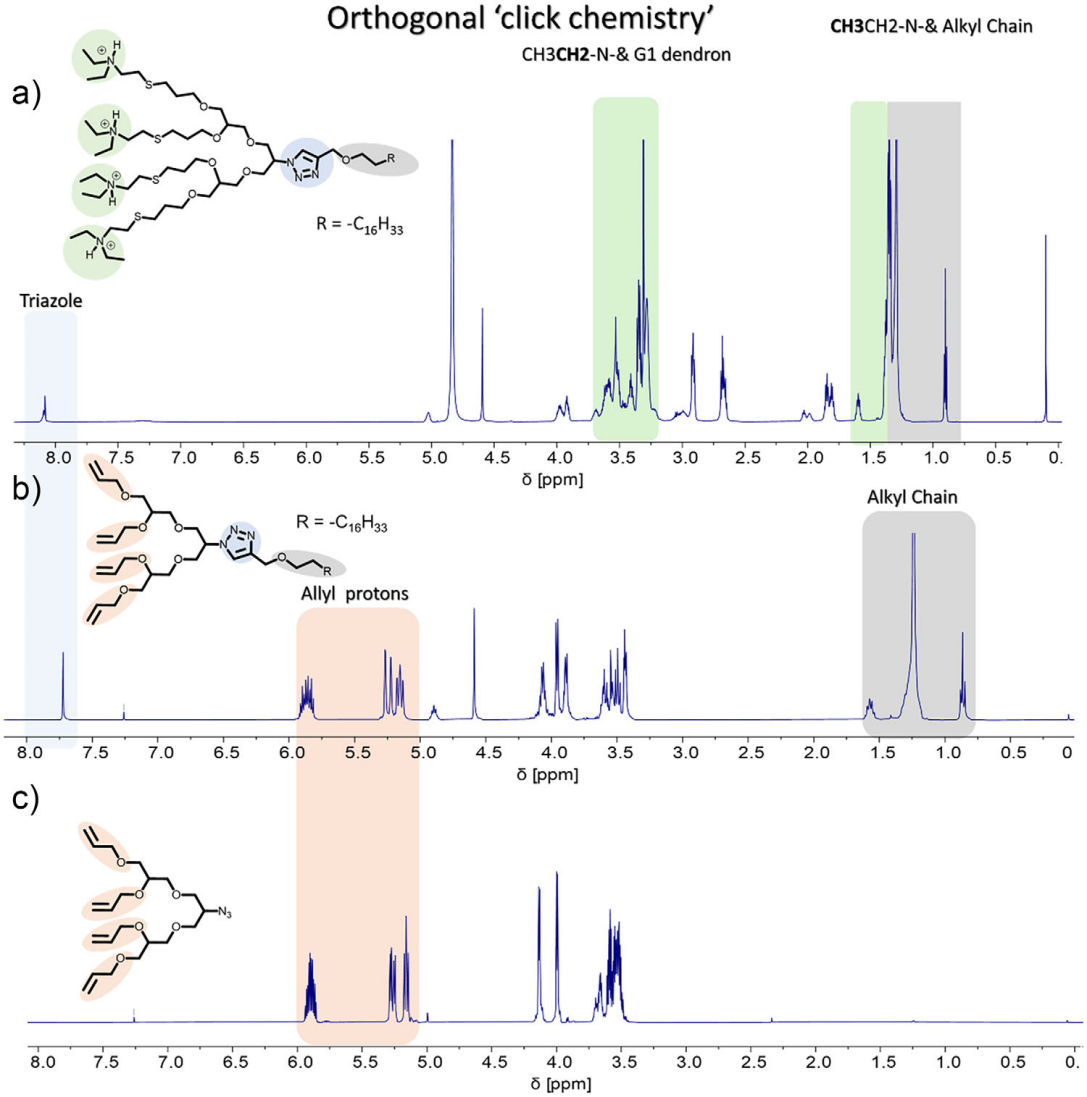
Spectral characterization of the orthogonal click chemistry approach used to synthesize cationic oligo‐glycerol surfactants: a) Thiol‐ene click reaction, indicated by the disappearance of allyl protons between δ 6.0 and 5.0 ppm.; b) Azide‐alkyne click reaction, confirmed by the appearance of the triazole proton peak at δ 7.7 ppm.; C) ¹H NMR spectrum of 4‐allyl‐G1‐N₃, showing the characteristic allyl protons δ 6.0–5.0 ppm.

After the synthesis and its full validations using ^1^H and ^13^ C NMR of both clickable sites in oligo‐glycerols (G0, G1, and G2) (Figure 1 and Figures S12–S52), the hydrophobic C12/C18‐alkyne was clicked onto the G0/G1/G2‐azide using copper sulphate and sodium ascorbate. The click product was fully characterized by ^1^H NMR, with the appearance of the triazole peak at 7.72 ppm (Figure 1b) and allyl protons in the range of 5.13–5.91 ppm (Figure 1b,c). These final monomers were also characterized with ESI‐MS (Supporting Information Experimental sections). The final thiol‐ene click reaction was carried out on all terminal allyl groups using a LED lamp (365 nm), with 2‐diethylaminoethanethiol hydrochloride as the cationic linker and 2,2‐dimethoxy‐2‐phenylacetophenone (DMPA) as the photoinitiator. Complete conversion of the thiol‐ene reaction was confirmed by the disappearance of allyl protons (5.13–5.91 ppm) in the ^1^H‐NMR spectrum (Figure 1a) and the appearance of the methyl and methylene protons of the tertiary amine group (Figure 1a). The final products were purified by dialysis over 2 days. These cationic surfactants were thoroughly characterized using various spectroscopic techniques, i.e., ^1^H and ^13^C NMR, ESI‐MS, to confirm their intended structures (Figures S12–S52). These novel synthesized surfactants were subjected to further physicochemical characterizations, i.e., zeta potential, size distribution by dynamic light scattering (DLS), and critical micelle concentration (CMC) by fluorescence spectroscopy using Nile Red as a probe (Table S1 and Figures S10, S11). The DLS data reveals that all the synthesized surfactants form micellar structures between 6 and 10 nm above their CMC value. Further, the zeta potential values demonstrate the availability of positive charges on the head groups of these surfactants (Table S1). After performing and validating the structural and physicochemical characterization, we subjected these surfactants to in‐vitro analysis to determine their antibacterial properties.

### In‐Vitro Antibacterial Activities

To better understand the correlation between the surfactants’ design and their antibacterial potential, the standard microdilution assay was performed using two distinct bacterial strains. Specifically, all six surfactants were tested at different concentrations for their potential to inhibit the bacterial growth of gram‐negative *E. coli* and gram‐positive MRSA.^[^ ^51^ ^]^ All compounds were tested for their antibacterial potential at concentrations below their CMC value (Figure S10 and Table S1).

We found that increasing the number of positively charged functional groups from 2 to 4 and decreasing the alkyl chain length from 18 to 12 carbon atoms significantly increased the inhibition potential against *E. coli* and MRSA. For the surfactant with a C18 alkyl chain and two positively charged functional groups (C18‐G0), an inhibition of the bacterial growth of MRSA was observed up to a concentration of 125 µg mL.^−1^ The inhibition potential increases with the reduction of the alkyl chain from 18 to 12 carbon atoms.

**Figure 2:**
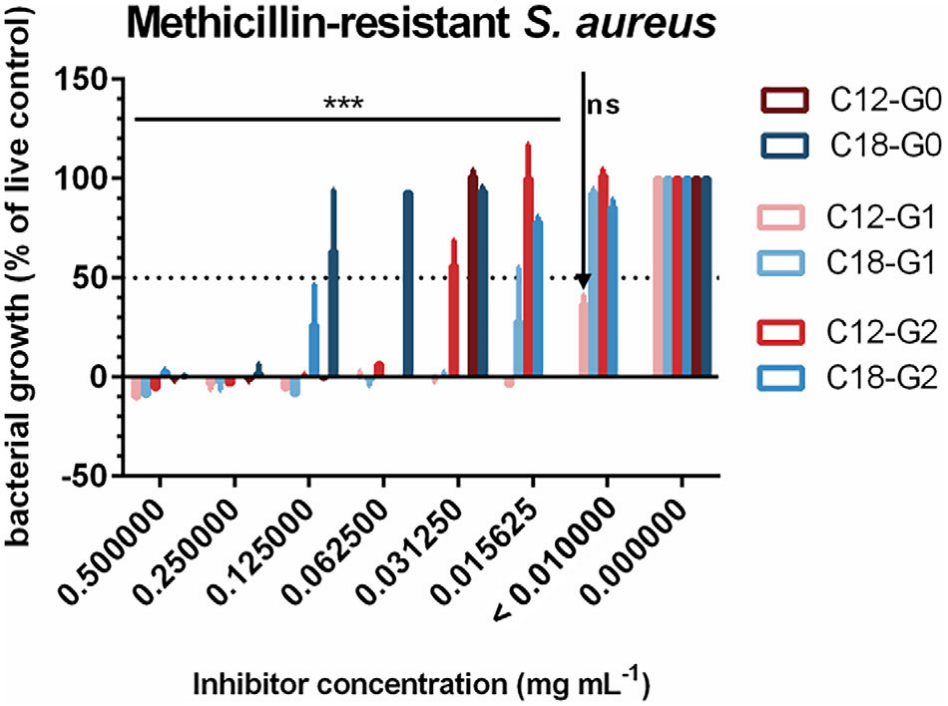
Bacterial growth (%) of MRSA against the inhibitor concentrations (mg mL^−1^) of the cationic surfactant (mean ± SD, *n* ≥ 2, statistics were determined using two‐way ANOVA, ***p < 0.001, ns refers to no significance, compared to untreated control which refers to an inhibitor concentration of 0.00 mg mL^−1^) after 24 h treatment.

The surfactant with two positively charged functional groups and a C12 alkyl chain (C12‐G0) showed inhibition of bacterial growth up to the final concentration of 31.3 µg mL^−1^. Increasing the number of positively charged functional groups caused an increase in the antibacterial potential against MRSA. In case of the G2 series, the bacterial growth increased at very low concentrations of 15.6 µg mL^−1^. In case of the G1 series, a difference in the inhibition potential was observed depending on the length of the alkyl chain. Here, the surfactant C18‐G1 showed inhibition potential up to a concentration of 15.6 µg mL.^−1^ The cationic surfactant C12‐G1 inhibited bacterial growth of drug‐resistant MRSA even below a concentration of 10.0 µg mL^−1^ (Figure 2).

**Figure 3:**
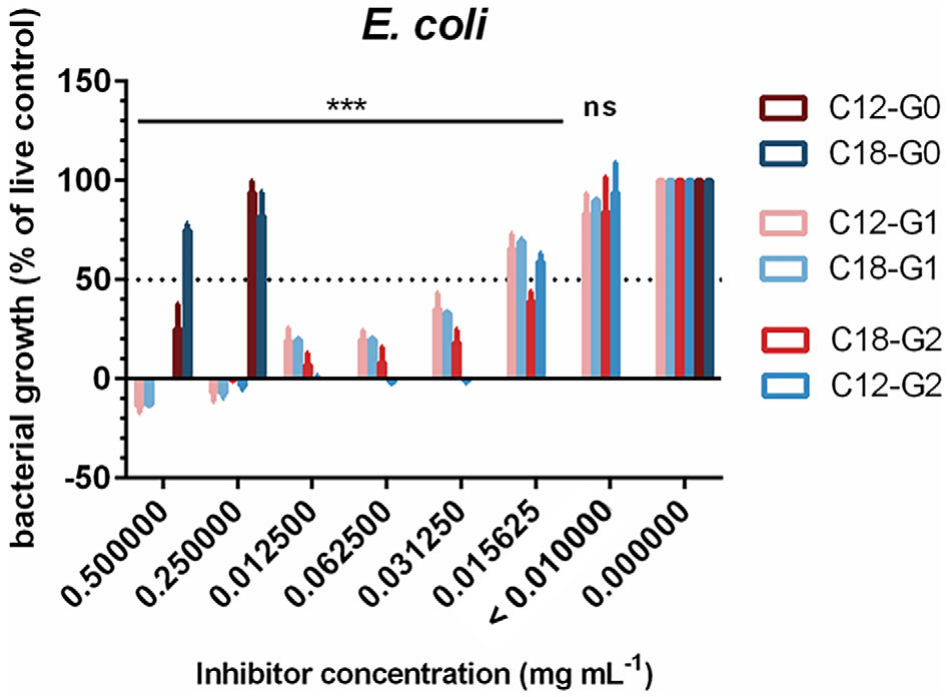
Bacterial growth (%) of *E. coli* against the inhibitor concentrations (mg mL^−1^) of the cationic surfactant (mean ± SD, *n* ≥ 2, statistics were determined using two‐way ANOVA, ***p < 0.001, ns refers to no significance, compared to untreated control which refers to an inhibitor concentration of 0.00 mg mL^−1^) after 24 h treatment.

As seen in Figure 3, the surfactant C18‐G0 was shown to be inactive against *E. coli*, as even at very high concentrations (500 µg mL^−1^) no inhibition of bacterial growth was visible. By contrast, replacing the C18 alkyl chain with a C12 alkyl chain conferred a moderate bactericidal activity against *E. coli*, with inhibition of bacterial growth observed at the high concentration of 500 µg mL^−1^. As the number of positively charged functional groups was increased from 2 to 4 and 8, the antibacterial potential of the surfactant increased. A similar inhibition potential was observed for the G1 and G2 series, whose four surfactants were shown to inhibit bacterial growth only up to very low concentrations of 15.6 µg mL^−1^ (Figure 3).

In summary, the antimicrobial properties of the surfactants are significantly influenced by the length of their alkyl chain and by their number of cationic groups. Variations in alkyl chain length and cationic group density affect the affinity of the surfactants for the bacterial surfaces, thereby altering bacterial binding efficiency. Overall, our cationic surfactants demonstrated stronger inhibition potential against gram‐positive MRSA; this observation can be explained by the difference in cell structure between gram‐positive and gram‐negative bacteria. The bacterial membrane consists of lipopolysaccharides, which include negatively charged lipids like phosphatidyl glycerol and cardiolipin. *S. aureus* contains a much higher amount of negatively charged lipids than *E. coli*, and so its electrostatic interaction with the cationic surfactant is much higher, resulting in an enhanced antibacterial effect.^[^ ^52^ ^]^

For both strains, an increase in the antibacterial effect was found upon increasing the number of positively charged functional groups from 2 to 4. This trend can be explained by the positively charged functional groups’ effect on the bacteria: Due to electrostatic interactions between the surfactant and the bacteria, the surfactant can adhere to the bacteria's surface.^[^ ^52^ ^]^ Similarly, a higher number of positively charged functional groups enhances the electrostatic interaction between the positively charged surfactant and the negatively charged bacteria, strengthening the surfactant's antibacterial effect.^[^ ^19^ ^]^ Contrary to our expectations, no increase in the antibacterial effect was conferred by increasing the number of positively charged functional groups from 4 to 8. This observation can be explained by an imbalance between the hydrophobic and hydrophilic units of the surfactant; we suspect that a higher number of positively charged functional groups might increase the steric shielding effect between the surfactant and the bacteria. Taking the length of the alkyl chain into account, we found that decreasing this length from 18 to 12 carbon atoms enhanced the antibacterial effect. As reported in the literature, a longer alkyl chain may fold back, thereby causing an imbalance between the hydrophobic and hydrophilic units of the surfactant and inducing an increased steric shielding effect toward the bacteria.^[^ ^33^ ^]^ Based on these results, we selected the surfactant with a C12 alkyl chain and an enhanced number of four cationic groups as the prime candidate.

**Figure 4:**
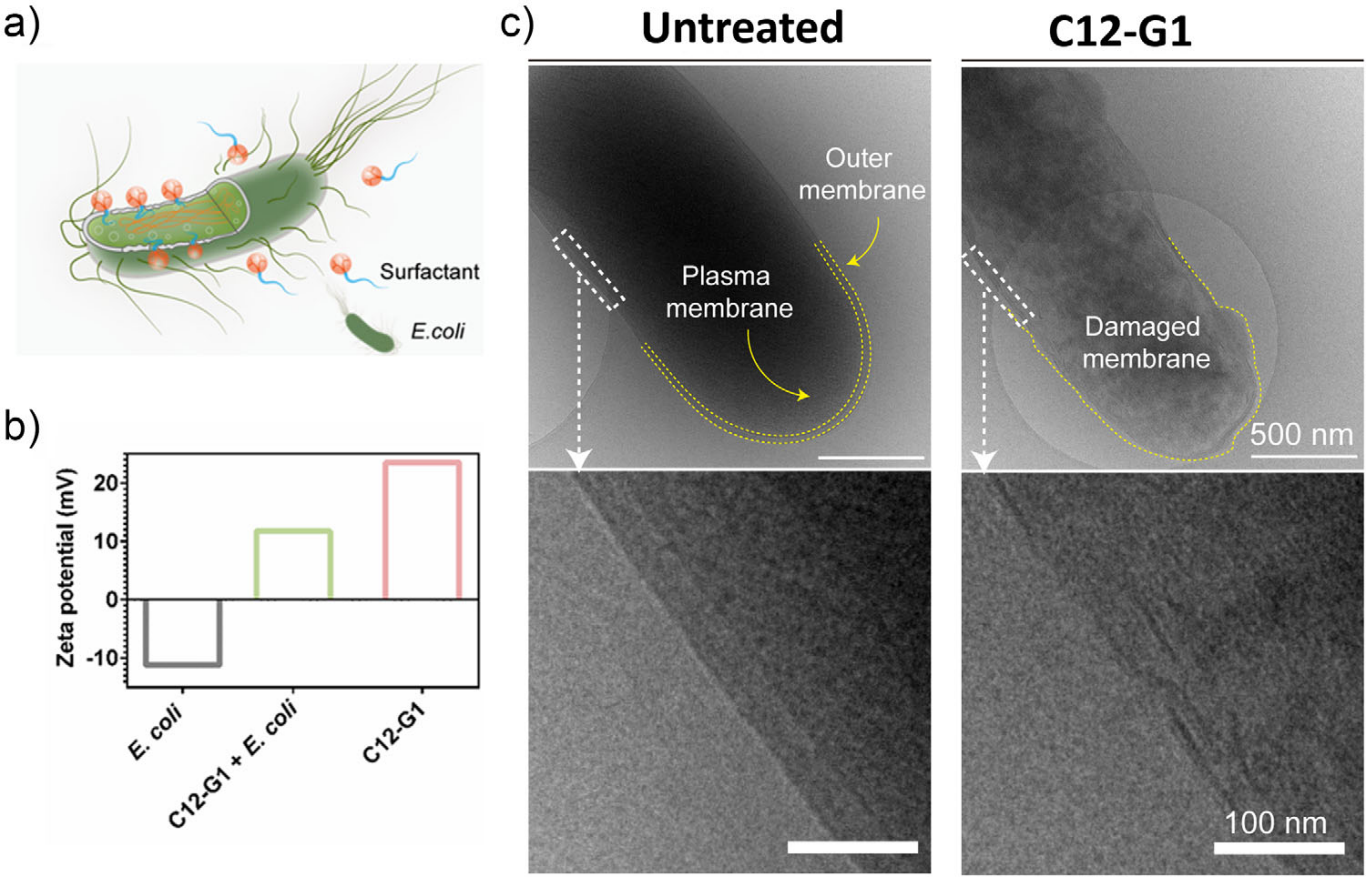
a) Schematic representation of the antibacterial mechanism by surfactants, b) zeta potential measurement of *E. coli*, C12‐G1 and *E. coli *+ C12‐G1, and c) representative Cryo‐TEM images of C12‐G1 and *E coli* (scale bar: 500 nm top, scale bar: 100 nm bottom).

For the next phase of our research, we were specifically interested in developing a more detailed understanding of this proposed electrostatic mechanism of action (Figure 4a). We therefore performed zeta potential measurements with the aim of proving the electrostatic interactions of the positively charged surfactant with the negatively charged bacteria. Cryo‐TEM images were performed to prove the disruption of the bacteria cell surface by these synthesized surfactants.

The surface charge of C12‐G1 was evaluated by measuring the zeta potential. Analysis revealed a zeta potential of *ζ* = 23.6 mV, indicating a positively charged surface. As prior literature has established, bacterial cells are negatively charged.^[^ ^53^ ^]^ As expected, we found a negative zeta potential for *E. coli* (*ζ* = −11.3 mV). Mixing the surfactant and bacteria should therefore cause the positively charged surfactant to adhere to the surface of the negatively charged bacterial cell. This adhesion should neutralize the negative charge of the bacteria, resulting in a neutral or positive charge for the bacteria‐surfactant complex. Indeed, we observed an overall positive charge (*ζ* = 11.8 mV) for the bacteria‐surfactant complex, further demonstrating the surfactant's ability to adhere electrostatically to the bacterial cell surface (Figure 4b). As illustrated in Figure 4a, upon adherence, the surfactant's hydrophobic alkyl chains anchor themselves in the bacterial cell wall, bringing about its destruction and ultimately cell death. In order to prove the destruction of the cell wall, cryo‐TEM images of treated and untreated bacteria were taken. Bacteria at a concentration of 10^8^ CFU mL^−1^ were treated with the cationic surfactant C12‐G1 for 2 h at a concentration of 5.00 µg mL^−1^. Afterward, Cryo‐TEM images were taken, revealing a strong difference between the untreated and the treated bacteria (Figure 4c). For the treated bacteria, a clear disruption of the outer membrane and the leakage of inner cell compartments can be observed.

**Figure 5:**
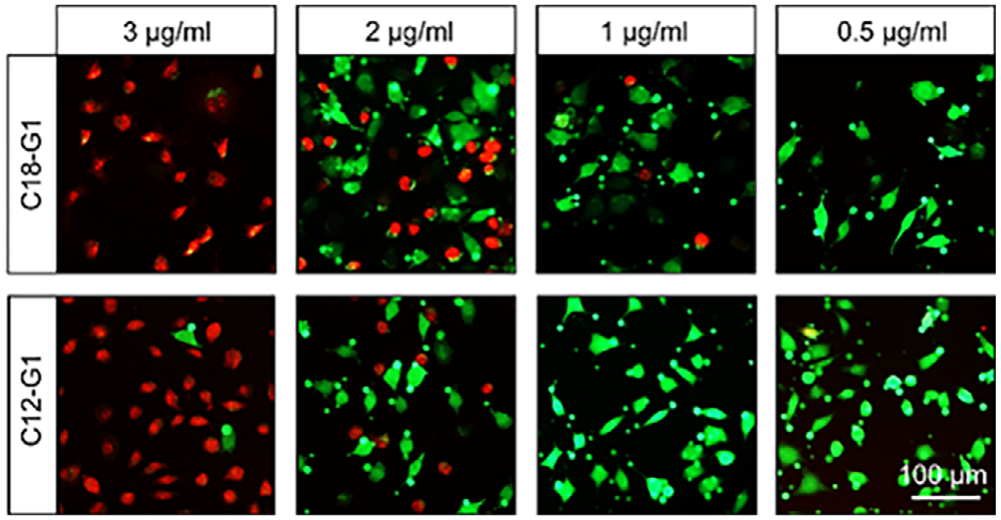
Representative live/dead cell staining (green: live, red: dead) for L929 cell lines. Scale bar = 100 µm.

The mechanism of cell wall disruption described here, caused by the alkyl chain of the surfactant, is nonselective with regard to the targeted cell type. This mechanism can therefore act not only on bacteria but also on eukaryotic cells. Indeed, most QACs are known to exhibit a cytotoxic effect toward mammalian cells.^[^ ^23^, ^24^ ^]^ To gain a better understanding of the cytotoxic effect of the synthesized cationic surfactants, we performed standard cell viability assays and live/dead staining. For all surfactants, the cytotoxic effect was significantly reduced at a concentration of 3.00 µg mL^−1^ (Figures S4–S9) as compared to higher concentrations. As the goal of our study was to investigate these cationic surfactants’ potential to promote wound healing, we were especially interested in investigating in detail the cytotoxic effect of the prime candidate, which belongs to the G1 series. Therefore, we took fluorescence microscopy images of L929 cells treated with C12‐G1 and C18‐G1 at different concentrations. As seen in Figure 5, a lowered cytotoxic effect was observed for very low concentrations of 2.00–1.00 µg mL^−1^.

**Figure 6:**
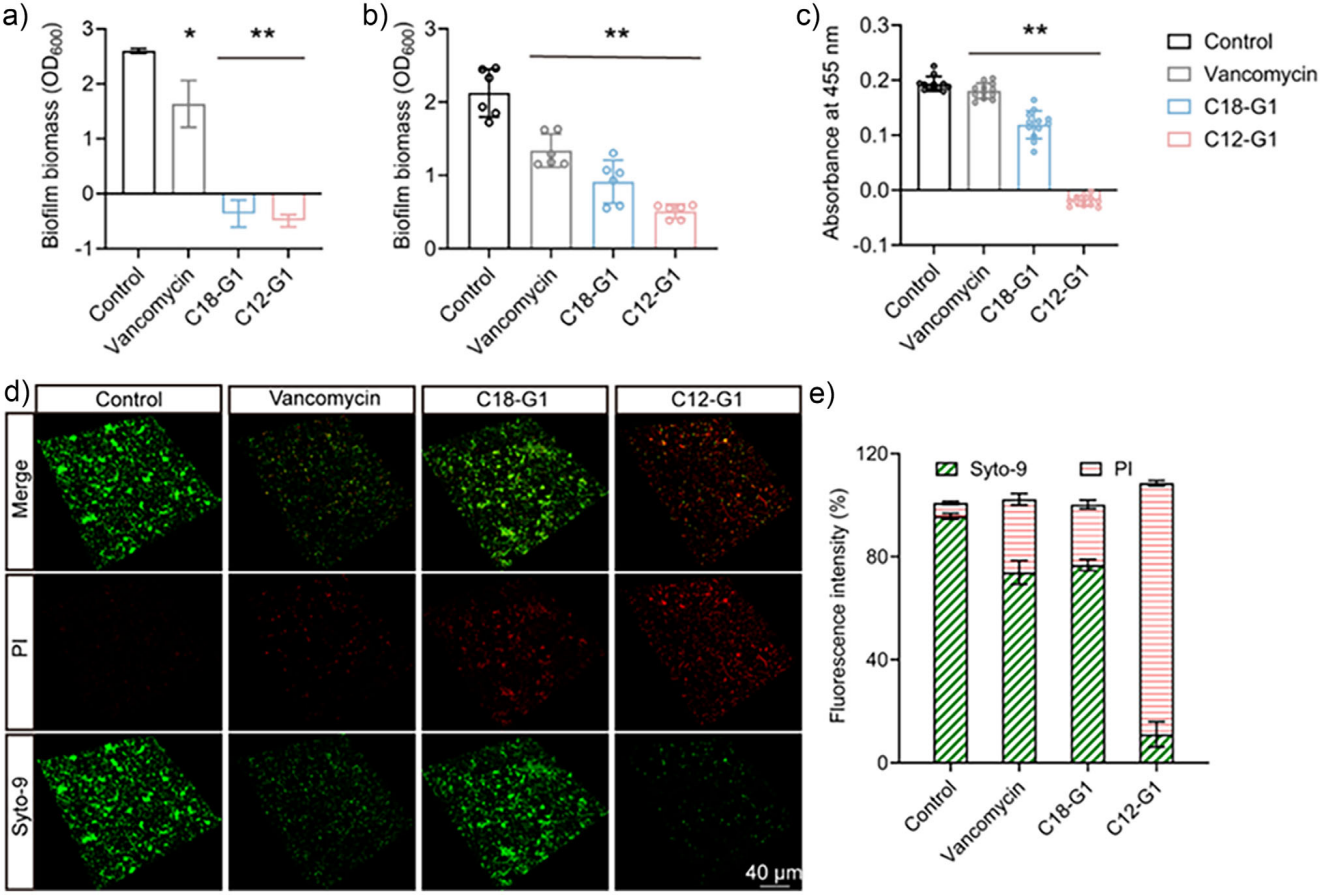
a) Biofilm formation after 72 h with various samples, b) mature biofilm inhibition after 24 h treatment with various samples, c) biofilm viability after 24 h treatment with various samples, d) representative live/dead bacterial staining of MRSA with various treatment after 24 h (green: live, red: dead, scale bar = 40 µm), and e) related statistics for fluorescence intensity, all experimental data are displayed as the average values (mean ± SD, *n* = 3). Data were analyzed for significance by two‐way ANOVA, and asterisks indicate significant differences compared with control (0.01<*p<0.05, 0.001<**p <0.01).

Encouraged by the antibacterial activity of our cationic surfactants, we further studied their antibiofilm performance at very low concentrations of 1.00 µg mL^−1^. We first set out to investigate the ability of C18‐G1 and C12‐G1 to inhibit biofilm formation. We simultaneously seeded MRSA in suspension with varying surfactants (1 µg mL^−1^ C12‐G1, 1.00 µg mL^−1^ C18‐G1, 16 µg mL^−1^ vancomycin, or none) before incubating the suspensions for 72 h. Both C18‐G1 and C12‐G1 prevented biofilm formation due to their robust planktonic MRSA‐killing ability (Figure 6a). We further tested the surfactants’ ability to eradicate mature biofilm. Briefly, mature biofilms were cultured from MRSA suspension for 72 h. Then different surfactants were added to the culture for another 24 h, followed by crystal violet staining. As shown in Figure 6b, we observed clear biofilm biomass reduction for the groups treated with C12‐G1 and C18‐G1. Quantitatively, at 1 µg mL^−1^, C12‐G1 and C18G1 removed 76.0% and 57.0% of biofilm, respectively. The slightly better biofilm eradication activity of C12‐G1 might be due to its higher penetration in the biofilm; C18‐G1 could be trapped by the biofilm's extracellular polymeric substances (EPS), which consist of polysaccharides and proteins crosslinked by hydrophobic interactions.^[^ ^54^, ^55^, ^56^ ^]^

We then used WST assay and live/dead bacteria staining to study viability of MRSA in the biofilm. From the results shown in Figure 6d,e, we can conclude that C12‐G1 exhibited higher bactericidal activity than C18‐G1; this conclusion also supports our hypothesis that the shorter aliphatic chain samples penetrated the biofilm more efficiently than the longer ones. Noticeably, as seen in Figure 6b, C12‐G1 removed most of the MRSA biofilm within 24 h of its addition to the biofilm.

### In‐Vivo Wound Healing Model

We further evaluated the in‐vivo antibacterial activities of C12‐G1 in wound healing models. Before performing in‐vivo experiments, we evaluated the biocompatibility of C12‐G1 in L929 cells and blood. We noticed that 1.00 µg mL^−1^ C12‐G1 did not appear to induce L929 cell death or haemolytic effects (Figure 5 and Figure S1). Based on this information, we used 1.00 µg mL^−1^ as the standard dose for all in‐vivo experiments. After an 8 mm skin defect was created on the mice and left for 24 h, MRSA dispersions (50 µL, 1 × 10^6 ^CFU/mL) were applied to the wound area. Then, the mice were randomly divided into three groups as follows: i) untreated, ii) vancomycin, and iii) C12‐G1. Wound images were taken at 0, 1, 3, 8, and 14 days after treatment.

**Figure 7:**
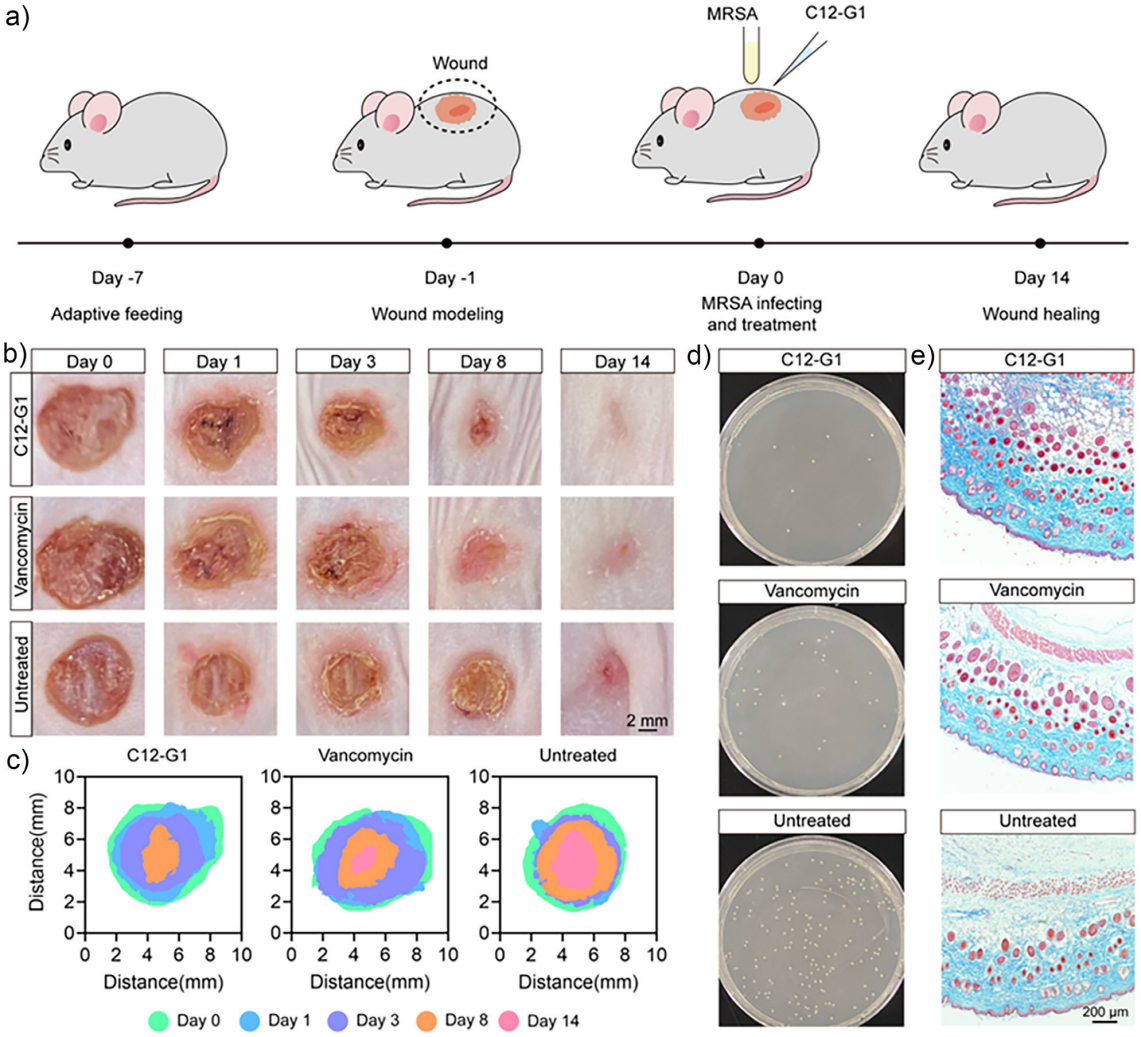
a) Schematic of the in‐vivo assay, b) representative images of the wound area from day 0 to day 14 (black scale bar = 2 mm), c) traces in the wound area from day 0 to day 14, d) agar plate culture of wound tissue 24  h after treatment, and e) representative images of Masson's trichrome staining (black scale bar = 200 µm).

As shown in Figures 7b,c, the C12‐G1 group exhibited the fastest recovery, achieving complete wound closure by day 14. By contrast, 54.5% and 13.6% of the wounds in the untreated group and the vancomycin group, respectively, remained unclosed.

Bacterial samples were collected from the wound by swabbing and titrated on agar plates, and a significant inhibition of MRSA growth was observed following C12‐G1 treatment (Figure 7d), which contributed to the accelerated wound healing. Since collagen is crucial for creating an optimal environment for cell growth and serves as a significant marker for tissue recovery, we used Masson's trichrome staining to evaluate collagen deposition during the wound‐healing process.^[^ ^57^ ^]^ The group treated with C12‐G1 showed the highest collagen deposition (Figure 7e and Figure S3), indicating optimal collagen recovery and accelerated tissue healing. To assess the safety profile, we recorded the body weight and survival rate of mice subjected to the different treatments. For the C12‐G1 treated group of mice the survival rate was 100% after 14 days of treatment and the body weight was slightly higher than that of the untreated group (Figure S2). In summary, C12‐G1 effectively clears MRSA infection in vivo and speeds up the healing of wounds. Furthermore, due to its appropriately sized lipophilic alkyl chain, C12‐G1 can effectively penetrate biofilms, providing a potential strategy to overcome bacterial resistance.

## Conclusion

We designed a library of six cationic surfactants with a different number of multivalent cationic charges and different length hydrophobic tails. In this study, we discovered that the surfactant design strongly correlates with their antibacterial efficacy. A proper balance between the hydrophobic and hydrophilic unit of the surfactant turned out to be decisive. We were able to identify one prime candidate C12‐G1 that compromises both a balanced ratio between the hydrophobic alkyl chain and the hydrophilic glycerol‐based unit which leads to an optimized antibacterial efficacy and a lowered cytotoxic effect. Further, these cationic surfactants have the potential to prevent biofilm formation and to eradicate already existing biofilms of drug‐resistant bacteria strains like MRSA. They effectively clear MRSA infection in vivo and speed up the healing of wounds which provides a potential strategy to overcome bacterial resistance.

## Supporting Information

Additional supporting information can be found online in the Supporting Information section at the end of this article.

## Conflict of Interests

The authors declare no conflict of interest.

## Supporting information



Supporting information

## Data Availability

The data that support the findings of this study are available in the supplementary material of this article.
