# Supplementary material for: Modular Synthesis of Dendritic Oligo‐Glycerol Cationic Surfactants for Enhanced Antibacterial Efficacy
Source: Angew Chem Int Ed Engl. 2025 Apr 14;64(22):e202425069. doi: 10.1002/anie.202425069 (PMC12105686; doi:10.1002/anie.202425069)
Supplement: Supplementary file 1 — Supporting information [file ANIE-64-e202425069-s001.docx]

Supporting Information

Modular Synthesis of Dendritic Oligo-glycerol Cationic Surfactants for Enhanced Antibacterial Efficacy

Natalie Hanheiser,^ǂ[a]^ Yuhang Jiang,^ǂ[a]^ Christian Zoister,^[a]^ Mathias Dimde,^[b]^ Katharina Achazi,^[a]^ Chuanxiong Nie,^[a]^ Yuanyuan Li,*^[c]^ Rainer Haag,*^[a]^ and Abhishek K. Singh*^[a]^

**Reagents**

All solvents were used in HPLC quality, as well as deuterated solvents. All reagents and solvents were used as purchased from commercial suppliers, without any prior purification. All reactions which involved air- or water-sensitive compounds were carried out in a dried flask under an argon atmosphere.

**Thin layer chromatography**

Thin layer chromatography (TLC) analysis was performed on Merck silica gel 60, Fluorescenceindikator F_254_. A solution of KMnO4 was used as a staining solution. The solution contains 1.5 g of KMnO_4_, 10 g K_2_CO_3_, and 1.25 mL 10% NaOH in 200mL water.

**Column chromatography**

The preparative purification of mixtures by column chromatography was conducted on Merck silica gel 60 (0.040–0.063 mm). The different eluents used are described in the synthesis section.

**NMR spectroscopy**

^1^H and ^13^C NMR spectra were recorded on Bruker Ultrashield 400 MHz spectrometers (400 MHz for ^1^H NMR; 100 MHz for ^13^C NMR) or JEOL Spectrometer ECZ600 (600 MHz for ^1^H NMR, 151 MHz for ^13^C NMR) or JEOL Spectrometer ECP500 (500 MHz for ^1^H NMR, 151 MHz for ^13^C NMR). Chemical shifts δ are given in ppm relative to TMS as an internal standard or relative to the resonance of the solvent (^1^H NMR: Chloroform: δ = 7.26 ppm; D_2_O: δ = 4.79 ppm; MeOD δ = 3.31 ppm; ^13^C NMR: Chloroform: δ = 77.00 ppm; MeOD: δ = 49.00 ppm). Deuterated solvents used are indicated in each case. Peak multiplicities are abbreviated as s: singlet; d: doublet; m: multiplet; t: triplet.

**Critical micelle concentration (CMC)**

The critical micelle concentration was determined by the fluorescence technique using the encapsulation of ‘Nile red’ as a model dye. A stock solution of the dye in THF (1 mg/mL) was prepared. Each 20 μL of this stock solution were added to 10 sample vials where the THF was allowed to evaporate to leave a thin film of the dye. 2.5 mg/mL stock solutions of the amphiphiles in Milli-Q water were stirred overnight. The dilution series of the amphiphile were then transferred to the dye loaded vials and kept stirring overnight. The non-encapsulated dye was removed by filtration through 0.45 μm PTFE filter. Fluorescence measurements were performed using a Cary Eclipse fluorescence spectrophotometer. The determination of the CMC was performed by plotting the fluorescence intensity at λ = 635 nm against the logarithm of the amphiphile concentration.

**DLS and Zetapotential**

Dynamic light scattering (DLS) measurements of size and size distribution were measured using a Malvern Zetasizer Ultra (Malvern Instruments Limited, U.K.) at a constant temperature of 25 °C PBS buffer solution at a fixed concentration of 1 mg/mL.

**Zetapotential-measurement Bacteria**

The bacteria strain *E. coli* DH5α was streaked out on an LB agar‑plate and incubated over night at 37 °C. The next day one colony was picked and diluted in 5.00 mL LB‑medium. After incubation over night at 37 °C and 250 rpm the bacteria solution was transferred to a 5.00 ml falcon tube and centrifuged at room temperature for 8 min at 4500 rcf. The bacteria pellet was resuspended in fresh LB‑medium and the concentration of bacteria cells in solution was adjusted to c = 10^6^-10^7^ CFU/mL for both bacteria strains. Compound C12-G1 was added in an 1:1 ratio to the bacteria solution at a concentration of 5.00 mg mL^-1^.

**Microdilution Assay**

The bacteria strains *E. coli* DH5α (Thermo Fisher) and MRSA (DMSZ No. 13661)^[^[^1^](#_ENREF_1)^]^ were streaked out on an LB agar‑plate and incubated over night at 37 °C. The next day one colony was picked and diluted in 5.00 mL LB‑medium. After incubation over night at 37 °C and 250 rpm the bacteria solution was transferred to a 5.00 ml falcon tube and centrifuged at room temperature for 8 min at 4500 rcf. The bacteria pellet was resuspended in fresh LB‑medium and the concentration of bacteria cells in solution was adjusted to c = 10^6^-10^7^ CFU/mL for both bacteria strains. Dilution rows of the cationic surfactants were prepared in LB‑medium on the 96‑well plate. Afterwards the bacteria solution was added to each well on the plate. As growth control bacteria without any treatment was added to the plate. As positive control 10% penicillin-streptomycin was added to the wells on the plate. A sterile control to exclude possible contaminations in the media was further included. The absorbance at 600 nm was measured before and after incubation overnight using a epoch2 plate‑reader. From comparing the absorbance before and after overnight incubation the bacterial growth was determined.

All measurements were performed with three technical and two or three biological repetitions. The bacterial growth was calculated by setting the bacterial growth without treatment to 100% and the positive control to 0%. All graphs were plotted using the GraphPad Prism 7 software.

**Cytotoxicity assessment**

For determining the cell viability a CCK-8 Kit was used following the manufacturers instructions.

HeLa (ATCC CCL-Z) cell was cultivated in RPMI‑medium. For cell viability measurements both cell lines were seeded in a 96‑well plate at a density of 5x10^4^ cells/mL in RPMI-Medium or DMEM‑Medium (90.0 μL/well). The seeded cells were incubated over night at 37°C and 5% CO_2_. A 1:3 dilution series of each compound was prepared starting with a stock concentration of 100.0 µg/mL. 10.0 μL/well of each compound was applied to the plate including negative (1% SDS) and positive (Medium, 10% DPBS) controls. The cells with compound were incubated for another 24 h at 37 °C and 5% CO_2_. After 24 h of incubation 10.0  μL/well of CCK-8 solution was added. After 3 h of incubation at 37 °C and 5% CO_2_ the absorbance was measured (450 nm/650 nm) using a Tecan SPARK Plate Reader.

All measurement were performed with two to three technical and three biological repeats. The cell viability was calculated by setting the positive control to 100% using the Excel software. All Graphs were plotted using GraphPad Prism 7.

**Live/dead assay**

L929 cells (DSMZ No. ACC 2) were seeded in 96-well plate (4000 cells/well), then 100 μL of complete medium was added to each well and incubated for 24 h. The medium was replaced with fresh medium containing C12-4Q or C18-4Q (3 μg/mL, 2 μg/mL, or 1 μg/mL) and culture for another 24 h. Then, cells were washed with PBS 3 times to remove the culture medium, and 100 μL of Calcein AM work solution and 10 μL PI were added. The cells were stained for 15 min at room temperature and then washed with PBS buffer. The cells were then visualized by fluorescence microscopy (Axio Observer, Zeiss).

**Haemolysis assay**

Briefly, the PBS-diluted red blood cells (RBCs) were mixed with different samples (C12-4Q, C18-4Q) with a variety of concentrations (3 μg/mL, 2 μg/mL or 1 μg/mL) in centrifuge tubes. RBCs treated with 1% Triton X-100 were considered the positive control group, and PBS-treated RBCs were set as a negative control group. All groups were incubated at 37 ℃ with shaking for 1 h and then centrifuged at 6000 rpm for 3 min. Then, the absorbance of the supernatant was measured at 545 nm by microplate reader (Spark).

***In vitro* anti biofilm assay**

For the biofilm formation inhibition assay, 190 μL of MRSA solution (10^6^ CFU/mL, LB medium) was introduced to each well in 96-well plate. Afterward, for each well, 10 μL different sample solutions (final concentration: 1 μg/mL C12-4Q, 1 μg/mL C18-4Q, 16 μg/mL vancomycin or PBS) were added and incubated for 72 hours under 37 ℃. The PBS group was set as the control group. Then, all mediums were removed, washed 3 times with PBS, and stained with 0.1 % crystal violet for 15 min at room temperature. Therefore, the biofilms were washed with RO water to remove the excess dye. After drying at room temperature, 200 μL of ethanol was used to redissolved the crystal violet in the bottom of each well. Finally, the optical density of each well at 600 nm was analyzed by microplate reader (Epoch 2).

For the biofilm eradication assay, we introduced 200 μL of MRSA solution (10^6^ CFU/mL, LB medium) to each well in 96-well plate and cultured them in 37 ℃ incubator for 72 hours to harvest mature biofilm. Then, for each well, all mediums were replaced with 100 μL different sample solutions (1 μg/mL C12-4Q, 1 μg/mL C18-4Q, 16 μg/mL vancomycin or PBS) for another 24 hours culture and afterward, removed all solutions and test for biofilm biomass using the same method as above.

Then, we tested biofilm viability. Briefly, we used the same methods to get mature biofilms with different treatments. Then, 100 μL WST work solution was added to each well and cultured for 3 hours in a 37 ℃ incubator. The absorbance at 455 nm was tested by a plate reader (Epoch 2). For live/dead staining, the procedure provided by the manufacturer (L13152, Thermofisher) was followed, and fluorescence images were obtained by confocal microscopy (Leica).

***In vivo* animal experiments**

Male ICR mice (6-8 weeks) supplied by the Institute of Comparative Medicine of Yangzhou University were maintained in a pathogen‑free environment (25 ± 2 °C and 50 ± 5 % humidity) under a 12 h light/dark cycle with free access to food and water. All animal experiments were approved by the Animal Care Committee of Nanjing Tech University (No. 202440105-06) prior to commencement. After one week of adaptive feeding of male ICR mice (18–20 g, 5 weeks), full-thickness excisional cutaneous wounds were created on the backs of mice. After anesthetizing mice with 2% pentobarbital, the back hair was shaved, and the back skin was cleaned with saline. A hole punch was used to create a 8-mm in-diameter defect on the back of the mice. Then, MRSA suspension (1×10^6^ CFUs / mL) was dropped onto the wound area to cause infection. After adding MRSA suspension for 24 hours, the mice were randomly divided into three groups and treated with PBS, C12-4Q, and vancomycin.

After the skin defect infection model was established, a suspension of 50 μL C12-4Q (1 μg/mL), vancomycin (16 μg/mL), or PBS was dropped onto the wound center separately. The digital camera was used to take photos of the skin defects on the first, third, eight, and fourteenth day. Image J software was used for quantification analysis and tracking wound area. The wound healing efficiency was quantified by comparing the wound area at different times with the area on day 0. In addition to observing the antibacterial activities of C12-4Q, the mouse wound tissue with different treatments was collected on the second day, homogenized with 2 mL LB broth and diluted 1000 times with PBS, then spread 50 μL on LB agar and incubated at 37 ℃ for 24h, the number of CFUs on the nutrient agar plate was counted.

For histological analysis, the obtained mouse wound tissues were embedded in paraffin to obtain 5 μm paraffin sections. The collagen was stained with Masson's trichrome kit. The fluorescence microscope was used to obtain optical images of the sections. The intensity of the collagen-positive area was quantified by Image-Pro Plus 6.0 software.

**Statistical analysis**

The data analysis was done by using GraphPad Prism 7 software. All data are expressed as mean ± SD from triplicated experiments. Differences were analyzed by a one‑ or two‑way ANOVA. One-way ANOVA was used for Figure S4-S6 while a two‑way ANOVA was used for Figure 2, Figure 3, Figure 6A, 6B, 6C, 6E and Figure S3B. P < 0.05 was considered statistically significant.

**Cryo-transmission electron microscopy**

The preparation of the bacteria followed already reported literature.^[^[^2^](#_ENREF_2)^]^ Therefore the bacteria was adjusted to a final concentration of 10^8^ CFU mL^-1^ and the compound was added at a final test concentration of 5.00 µg mL^-1^.

Perforated carbon film-covered microscopical 200 mesh grid (R1/4 batch of Quantifoil, Micro Tools GmbH, Jena, Germany) were cleaned with chloroform and hydrophilised by 60 s glow discharging at 8 W in a BAL-TEC MED 020 device (Leice Microsystem, Wetzlar, Germany) before 5 µL aliquots of the sample solution were applied to the grids. The samples were automatically blotted and vitrified with FEI Vitrobot Mark IV (Thermo Fisher Scientific Inc., Waltham, Massachusetts, USA) using liquid ethane as cryogen. Cryo-TEM measurements were carried out on a Tecnai F20 TEM (FEI Company, Oregon) equipped with a field emission gun (FEG) at an acceleration voltage of 160 kV using a Gatan cryo holder at 94 K sample temperature. By using the microscope’s low-dose protocol, the micrographs were recorded with a FEI Eagle 4k X 4k CCD camera in twofold binning mode.


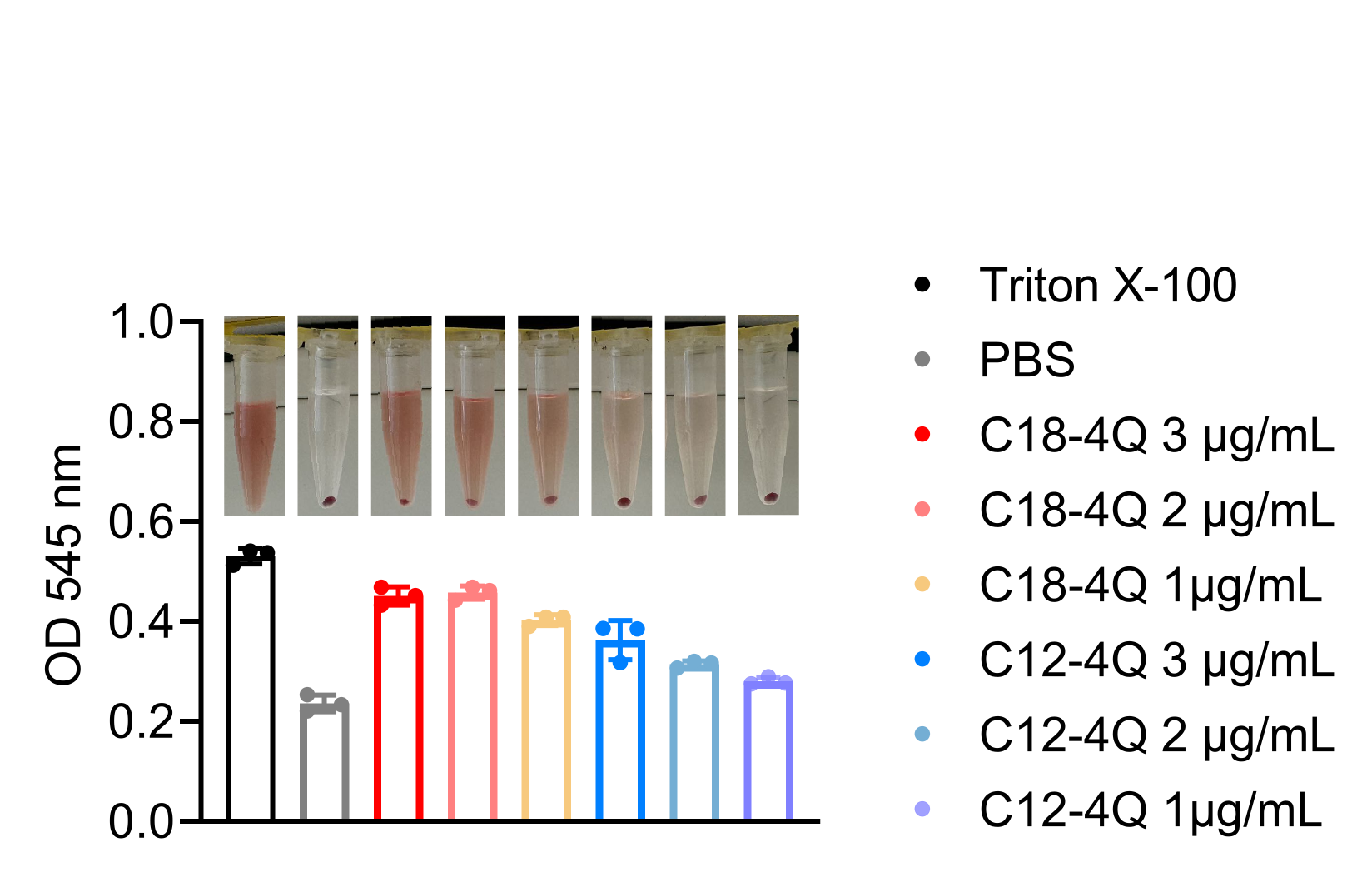


**Figure S1.** Hemolysis effect of C18-4Q and C12-4Q under different concentrations. Triton X‑100 treated red blood cells (RBCs) as positive control, and PBS treated RBCs as negative control (mean ± SD, n = 3).


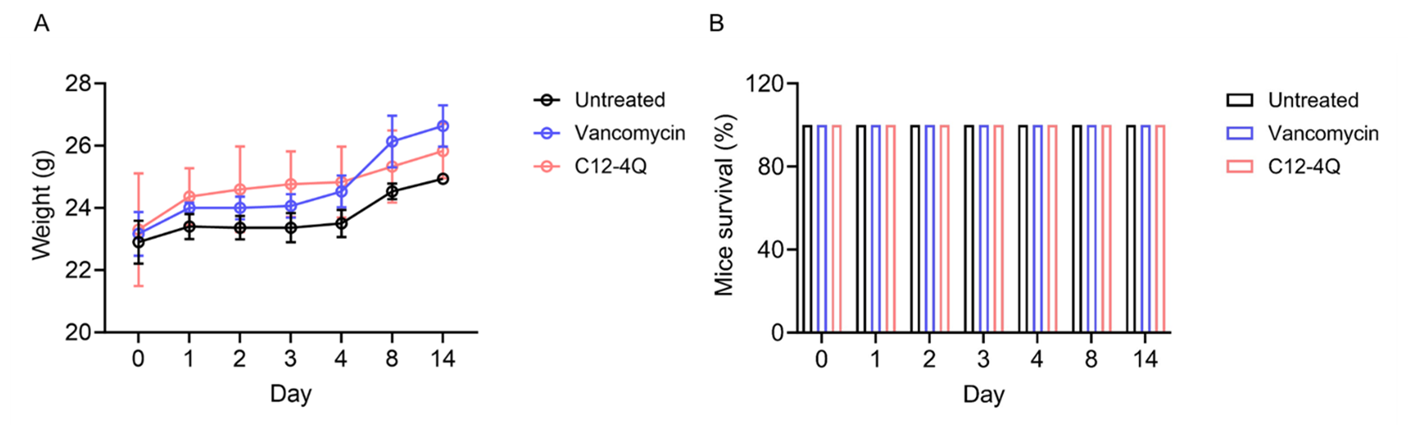


**Figure S2.** A) Mouse weight change curves during modeling (mean ± SD, n = 3). B) Mice survival rate during 14 days of treatment (mean ± SD, n = 3).

**Figure S3.** A) Statistics on wound area at different time points (mean ± SD, n = 3). B) Quantitative analysis of Masson’s trichrome staining (mean ± SD, n = 3, ***p*＜ 0.01, 0.01＜****p* ＜ 0.001, compared with untreated group).


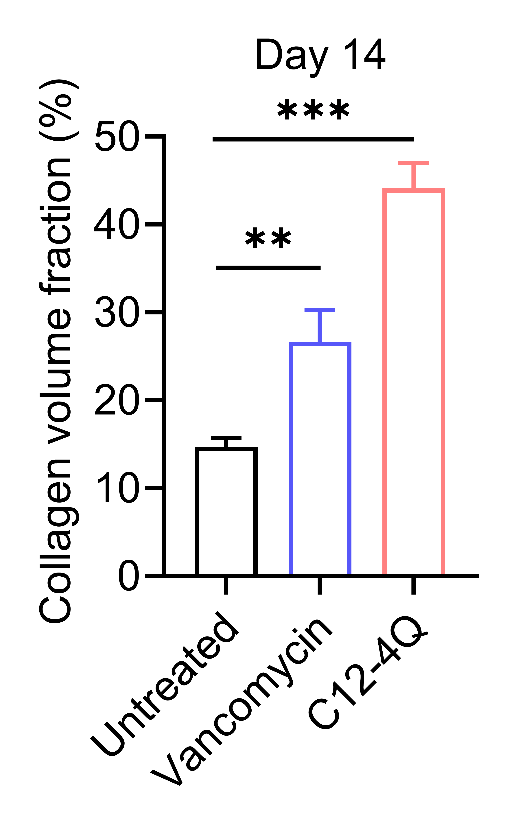

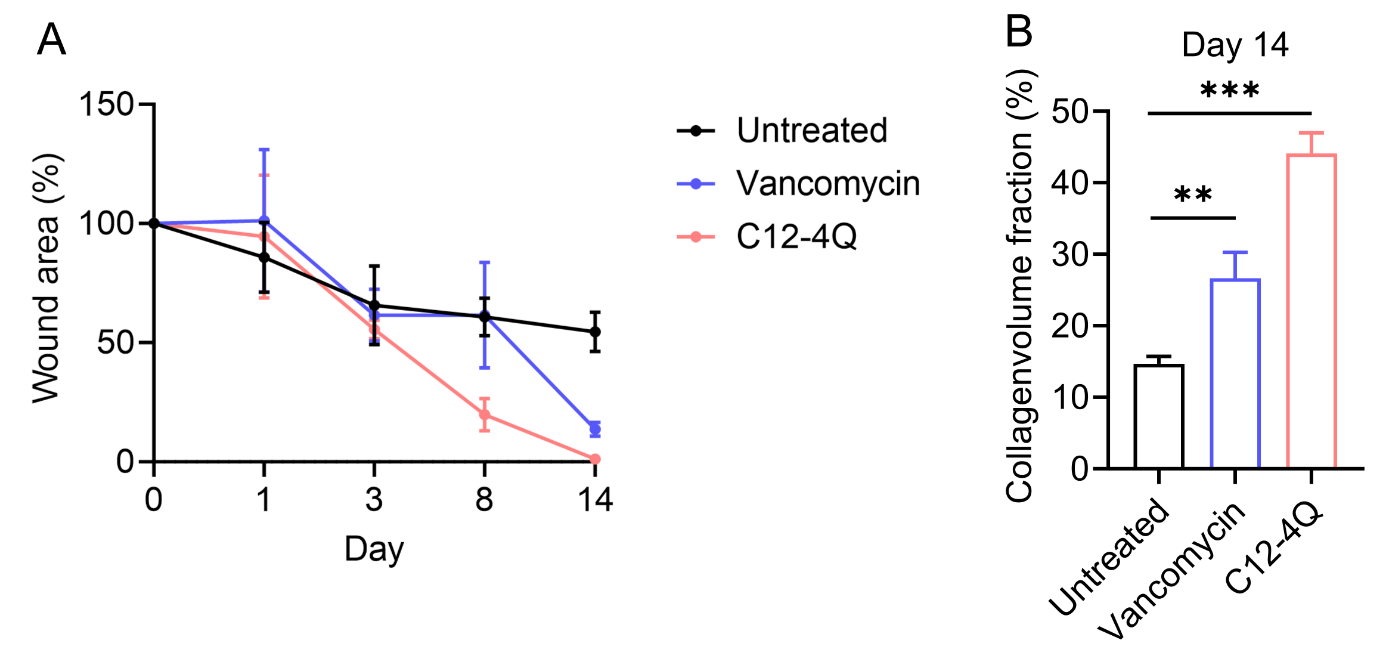


B





**Figure S4.** Cytotoxicity assessment of C12-G0 using HeLa cell line. Showing the cell viability (%) against different Inhibitor concentrations and PBS and SDS (1%) as positive and negative controls (mean ± SD, n = 3, statistics were determined using one‑way ANOVA, ***p < 0.001, compared to the negative control).





**Figure S5.** Cytotoxicity assessment of C18-G0 using HeLa cell line. Showing the cell viability (%) against different Inhibitor concentrations and PBS and SDS (1%) as positive and negative controls (mean ± SD, n = 3, statistics were determined using one‑way ANOVA, ***p < 0.001, ns refers to no significance, compared to the negative control).


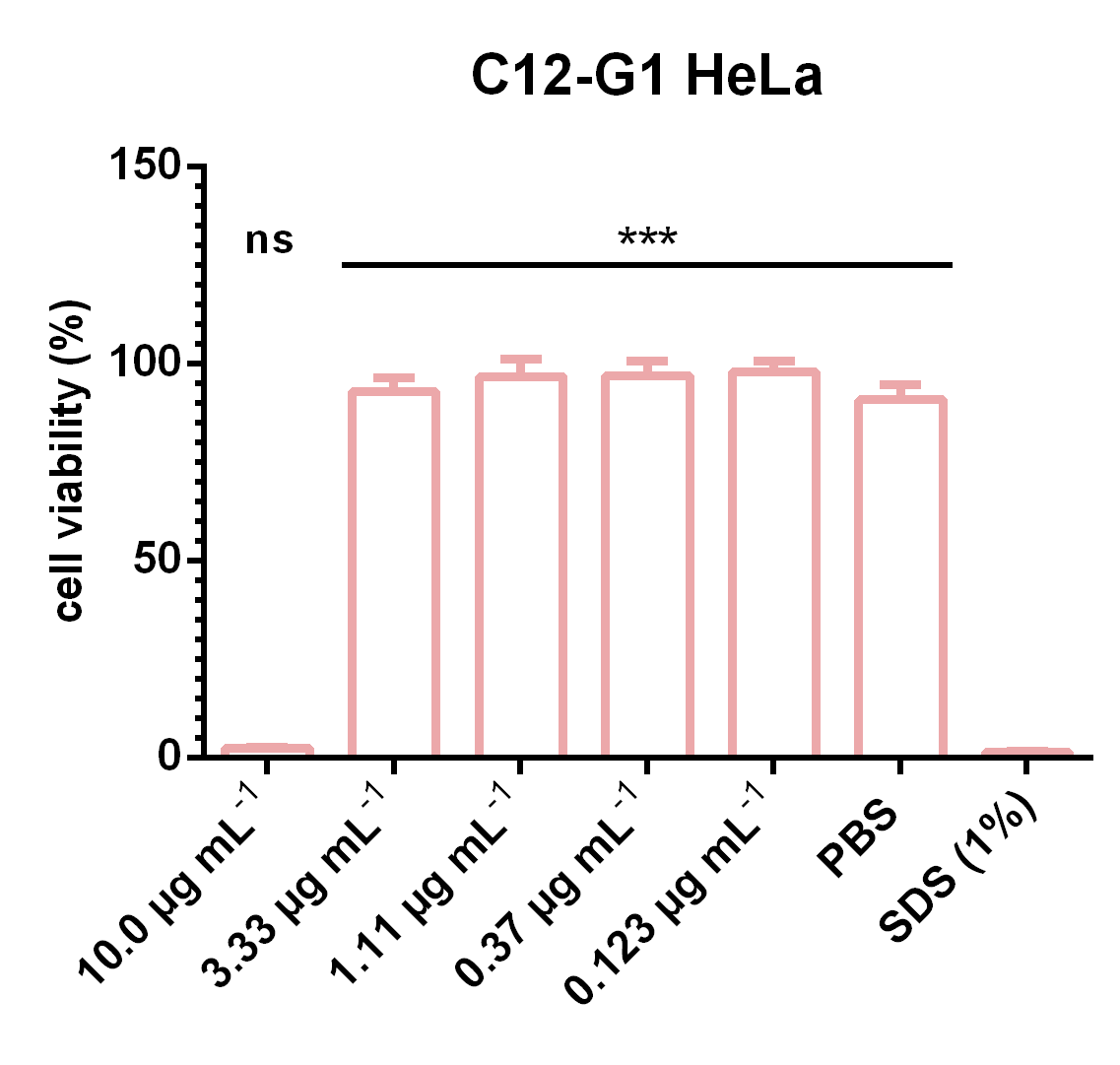


**Figure S6.** Cytotoxicity assessment of C12-G1 using HeLa cell line. Showing the cell viability (%) against different Inhibitor concentrations and PBS and SDS (1%) as positive and negative controls (mean ± SD, n = 3, statistics were determined using one‑way ANOVA, ***p < 0.001, ns refers to no significance, compared to the negative control).





**Figure S7.** Cytotoxicity assessment of C18-G1 using HeLa cell line. Showing the cell viability (%) against different Inhibitor concentrations and PBS and SDS (1%) as positive and negative controls (mean ± SD, n = 3, statistics were determined using one‑way ANOVA, ***p < 0.001, ns refers to no significance, compared to the negative control).





**Figure S8.** Cytotoxicity assessment of C12-G2 using HeLa cell line. Showing the cell viability (%) against different Inhibitor concentrations and PBS and SDS (1%) as positive and negative controls (mean ± SD, n = 3, statistics were determined using one‑way ANOVA, ***p < 0.001, compared to the negative control).





**Figure S9.** Cytotoxicity assessment of C18-G2 using HeLa cell line. Showing the cell viability (%) against different Inhibitor concentrations and PBS and SDS (1%) as positive and negative controls (mean ± SD, n = 3, statistics were determined using one‑way ANOVA, ***p < 0.001, compared to the negative control).

## Synthesis of individual compounds

#### C12-alkyne

NaH (2.0 eq, 1.30 g, 53.7 mmol) was dissolved under dry conditions and ice cooling in THF (50 mL). Dodecanol (1.0 eq, 5.03 g, 26.8 mmol) and propargyl bromide (1.2 eq, 1.81 g, 32.2 mmol) were added slowly to the reaction mixture. The reaction was carried out over night at 50 °C. The progress of the reaction was monitored using thin layer chromatography. The reaction was quenched by EtOH (10 mL). The solvents were removed under reduced pressure and the residue was extracted with DCM/H_2_O. The combined organic layers were dried over Na_2_SO_4_. The crude product was purified by column chromatography using hexane/EtOAc. The product (5.50 g, 91 %) was obtained as a colorless oil.

**^1^H NMR** (600 MHz, CHLOROFORM-*D*) δ 4.13 (d, *J* = 2.4 Hz, 2H), 3.50 (t, *J* = 6.7 Hz, 2H), 2.41 (t, *J* = 2.4 Hz, 1H), 1.61 – 1.58 (m, 2H), 1.36 – 1.23 (s, 18H), 0.88 (t, *J* = 7.0 Hz, 3H) ppm.

**^13^C NMR** (151 MHz, CHLOROFORM-*D*) δ 80.21, 74.15, 70.47, 58.14, 32.07, 29.80, 29.78, 29.73, 29.65, 29.59, 29.50, 26.23, 22.83, 14.25 ppm.

#### C18-alkyne

NaH (2.0 eq, 0.89 g, 37.0 mmol) was dissolved under dry conditions and ice cooling in THF (50 mL). Octadecanol (1.0 eq, 5.00 g, 18.5 mmol) and propagylbromide (1.2 eq, 1.30 g, 22.2 mmol) were added slowly to the reaction mixture. The reaction was carried out over night at 50 °C. The progress of the reaction was monitored using thin layer chromatography. The reaction was quenched by EtOH (10 mL). The solvents were removed under reduced pressure. The residue was extracted with DCM/H_2_O. The combined organic layers were dried over Na_2_SO_4_. The crude product was purified by column chromatography using hexane/EtOAc. The product (5.41 g, 95 %) was obtained as a colorless oil.

**^1^H NMR** (700 MHz, CDCl_3_) δ 4.13 (d, *J* = 2.4 Hz, 2H), 3.51 (t, *J* = 6.7 Hz, 2H), 2.41 (t, *J* = 2.4 Hz, 1H), 1.60 – 1.58 (m, 2H), 1.38 – 1.19 (m, 28H), 0.88 (t, *J* = 7.1 Hz, 3H) ppm.

**^13^C NMR** (176 MHz, CDCl_3_) δ 80.23, 77.34, 77.16, 76.98, 74.14, 70.48, 58.15, 32.09, 29.86, 29.82, 29.76, 29.74, 29.67, 29.60, 29.52, 26.25, 22.85, 14.27 ppm.

**MS** (ESI) m/z = 331.3107; [M+Na]^+^ (calculated for C_15_H_27_N_3_O_6_Na: 331.3079).

#### G0-OH

|  |
| --- |

Allyl glycidyl ether (1.00 eq., 5.00 g, 5.16 mL, 43.8 mmol) and allyl alcohol (2.00 eq., 5.09 g, 6.00 mL, 87.6 mmol) were dissolved in 20.0 mL H_2_O and 20.0 mL toluene. Afterwards, potassium hydroxide (2.00 eq., 0.25 g, 87.6 mmol) and tetrabutylammonium bromide (0.10 eq., 1.41 g, 4.38 mmol) was added to the reaction mixture. The reaction mixture was stirred over night at 50 °C. The crude product was purified by extraction using EtOAc/H_2_O. The combined organic phases were dried using Na_2_SO_4_ and further purified using distillation. G0-OH (4.67 g, 27,1 mmol, 62 %) was obtained as a slightly yellow liquid.

**^1^H NMR** (500 MHz, CD3OD, 25 °C): δ= 5.96-5.86 (m, 2H), 5.31-5.24 (m, 2H), 5.19-5.13 (m, 2H), 4.83 (s, 1H), 4.04-3.98 (m, 4H), 3.53-3.41 (m, 4H) ppm.

**^13^C NMR** (151 MHz, CD3OD): δ= 135.80, 116.85, 72.94, 72.39, 70.33 ppm.

**MS (ESI)** m/z = 195.1142; [M+Na]^+^ (calculated for C_9_H_16_O_3_: 195.0998).

#### G0-N_3_

|  |
| --- |

Under inert conditions, G0-OH (1.00 eq., 5.00 g, 29,1 mmol) was dissolved in 20.0 mL dry DCM followed by the dropwise addition of triethylamine (3.00 eq., 8.82 g, 12.1 mL, 87.2 mmol) and mesyl chloride (1.50 eq., 4,99 g, 3,37 mL, 43,6 mmol). The reaction process was monitored by thin layer chromatography in 30% EtOAc/hexane. After 3 h, the crude product was extracted using DCM/H_2_O. The obtained G1-OMs was dissolved in dry *N,N‑*dimethylformamide (30 mL). Sodium azide (3.00 eq., 5.67 g, 87.2 mmol) was added and the reaction mixture was stirred over night at 80°C. The reaction progress was monitored via thin layer chromatography using 30% EtOAc/hexane. The crude product was extracted using DCM/H_2_O. The combined organic phases were dried using Na_2_SO_4_ and purified by column chromatography using 15% EtOAc/hexane as eluent. The product G0-N3 (0.60 g, 3.04 mmol, 11 %) was obtained as a brown, oily liquid.

**^1^H NMR** (500 MHz, CD3OD, 25 °C): δ= 5.96-5.86 (m, 2H), 5.33-5.26 (m, 2H), 5.22‑5.15 (m, 2H), 4.06-3.97 (m, 4H), 3.62-3.57 (m, 2H), 3.56-3.50 (m, 2H) ppm.

**^13^C NMR** (151 MHz, CD3OD): δ= 135.64, 117.13, 73.02, 70.70, 61.97 ppm.

**MS** (ESI) m/z = 220.0977; [M+Na]^+^ (calculated for C_9_H_16_O_3_: 220.1098).

#### C12-G0

|  |
| --- |

C12-Allyl (1.20 eq., 0.60 g, 1.86 mmol) and G0-N3 (1.00 eq., 0.30 g, 1.52 mmol) were dissolved in 20.0 mL THF and 20.0 mL H_2_O. Copper sulphate and sodium ascorbate were dissolved in water separately and added to the reaction mixture simultaneously. After stirring over night at 50 °C the crude product was extracted using EtOAc/H_2_O, the combined organic phases were dried using Na_2_SO_4_ and the crude product was purified by column chromatography using 30.0 % EtOAc/hexane as eluent. C12-G0-2Aklyl (0.60 g, 1.32 mmol, 87 %) C12‑G0‑2Alkyl (1.00 eq., 0.60 g, 1.13 mmol) was dissolved in 20.0 mL MeOH. 2‑Diethylaminoethylethanethiol hydrochloride (3.00 eq., 0.46 g, 3.90 mmol) and 2,2‑Dimethoxy-2-phenylacetophenon (0.10 eq., 0.03 g, 0.113 mmol) were added to the reaction mixture. After stirring over night under UV-radiation at 365 nm the crude product was purified by dialysis in water for 48 h. The product C12-G0 (0.20 g, 0.28 mmol, 25 %) was obtained as a yellow solid.

**^1^H NMR** (500 MHz, CD3OD, 25 °C): δ= 8.02 (s, 1H), 4.56-4.51 (m, 2H), 3.91-3.78 (m, 3H), 3.58-3.50 (m, 1H), 3.50-3.43 (m, 3H), 3.29-3.25 (m, 6H), 3.20-3.07 (m, 10H), 2.80-2.72 (m,3H) 2.53-2.46 (m, 3H), 1.80-1.71 (m, 3H), 1.60-1.47 (m, 1H), 1.34-1.19 (m, 36H), 0.86 (t, J= 7.1 Hz, 3H) ppm.

**^13^C NMR** (151 MHz, CD3OD): δ= 57.05, 51.37, 48.86, 48.29, 48.15, 32.66, 31.49, 30.34, 28.81, 8.94 ppm.

**MS (ESI)** m/z = 704.4845; [M+H]^+^ (calculated for C_37_H_78_N_5_O_9_S_2_: 704.5578).

#### C18-G0

|  |
| --- |

C18-Allyl (1.20 eq., 0.59 g, 1.83 mmol) and G0-N3 (1.00 eq., 0.30 g, 1.52 mmol) were dissolved in 20.0 mL THF and 20.0 mL H_2_O. Copper sulphate and sodium ascorbate were separately dissolved in water and simultaneously added to the reaction mixture. After stirring over night at 50 °C the crude product was extracted using EtOAc/H_2_O, the combined organic phases were dried using Na_2_SO_4_ and purified by column chromatography using 30% EtOAc/hexane as eluent. C18-G0-2Alkyl (0.60 g, 1.15 mmol, 76%) was found as a colorless, slightly viscous liquid. C18-G0-2Alkyl (1.00 eq., 0.60 g, 1.15 mmol) was dissolved in 20.0 mL MeOH. 2-Diethylaminoethanethiol hydrochloride (3.00 eq., 0.410 g, 3.46 mmol) and 2,2-Dimethoxy-2-phenylacetophenon (0.10 eq., 30.0 mg, 0.115 mmol) was added to the reaction mixture. After stirring for 3 h under UV-radiation at 365 nm the crude product was purified by dialysis in water for 48 h. The final product C18‑G0 (0.40 g, 0.51 mmol, 44%) was obtained as a brown solid.

**^1^H NMR** (500 MHz, CD3OD): δ= 8.06 (s, 1H), 4.01-3.74 (m, 1H), 3.65-3.48 (m, 1H), 3.55-3.43 (m, 9H), 3.31-3.21 (m, 14H), 3.18-3.11 (m, 6H), 2.98-2.77 (m, 2H), 2.62-2.40 (m, 1H), 1.851.68 (m, 1H), 1.68-1.44 (m, 1H), 1.38-1.27 (m, 42H), 0.92-0.87 (m, 3H) ppm.

**^13^C NMR** (151 MHz, CD3OD): δ= 145.74, 124.66, 71.60, 70.62, 70.32, 64.53, 62.49, 52.59, 49.14, 48.86, 32.92, 30.63, 30.46, 29.14, 23.59, 14.30, 9.41 ppm.

**MS (ESI)** m/z = 786.5475; [M+H]^+^ (calculated for C_43_H_90_N_5_O_3_S_2_: 785.9927).

#### pG1-OH

Synthesized according to literature protocol.^[^[^3^](#_ENREF_3)^]^

**^1^H NMR** (500 MHz, MeOD) δ 4.26 (p, *J* = 5.7 Hz, 2H), 4.06-4.04 (m, 2H), 3.87-3.85 (m, 1H), 3.74-3.72 (m, 2H), 3.55-3.50 (m, 8H), 1.39 (s, 6H), 1.33 (s, 6H) ppm.

**^13^C NMR** (151 MHz, MeOD) δ 109.17, 74.84, 72.70, 72.65, 72.14, 69.29, 66.29, 25.80, 24.40 ppm.

**MS** (ESI) m/z = 343.1787; [M+Na]^+^ (calculated for: C_15_H_28_O_7_Na: 343.1835).

#### pG1-N3

pG1-OH (1.0 eq, 4.02 g, 12.5 mmol) was dissolved in DCM (50 mL). Under cooling with an ice bath, NEt_3_ (3.5 eq, 6.10 mL, 43.7 mmol) was added slowly. After 1 h of stirring MsCl (1.5 eq, 1.5 mL, 18.8 mmol) was added portion wise. The reaction was stirred over night at rt. The progress of the reaction was monitored by the thin layer chromatography using hexane/EtOAc. The crude product was extracted with DCM/H_2_O. The combined organic layers were dried with Na_2_SO_4_. The obtained pG1-OMs (1.0 eq, 5.30 g, 13.3 mmol) was dissolved in dry DMF (35 mL). NaN_3_ (3.0 eq, 2.61 g, 39.9 mmol) was added, the reaction mixture was heated up to 80 °C and stirred overnight. The progress of the reaction was monitored by thin layer chromatography using DCM/MeOH. The crude product was again extracted with DCM/H_2_O. The combined organic layers were dried with Na_2_SO_4_ and purified with column chromatography using DCM/MeOH as eluent. The product pG1-N_3_ (3.63 g, 81%) was obtained.

**^1^H NMR** (700 MHz, MeOD) δ 4.25 (tt, *J* = 6.4, 5.3 Hz, 2H), 4.05 (m, 2H), 3.77 – 3.69 (m, 3H), 3.68 – 3.63 (m, 2H), 3.61 – 3.51 (m, 6H), 1.39 (s, 6H), 1.33 (s, 6H) ppm.

**^13^C NMR** (176 MHz, MeOD) δ 110.52, 76.10, 76.08, 73.21, 73.16, 72.17, 72.07, 67.46, 62.05, 61.98, 61.91, 27.03, 25.66 ppm.

**MS** (ESI) m/z = 368.18; [M+Na]^+^ (calculated for C_15_H_27_N_3_O_6_Na: 368.19).

#### G1-N3

pG1-N3 (1.0 eq, 3.50 g, 10.1 mmol) was dissolved in MeOH. Dowex 50WX8 (50 w%, 1.75 g) and water (0.1 mL) were added to the reaction mixture. The reaction was carried out over night at 50 °C and monitored by thin layer chromatography using DCM/MeOH. After completion of the reaction, the Dowex was removed by filtration, the solvent was removed under reduced pressure and the product (2.52 g) was obtained with a yield of 93 %.

**^1^H NMR** (500 MHz, MeOD) δ 3.78 – 3.73 (m, 3H), 3.66 – 3.44 (m, 12H) ppm.

**^13^C NMR** (126 MHz, MeOD) δ 73.78, 73.73, 72.19, 72.15, 72.07, 64.35, 64.34, 62.03 ppm.

**MS** (ESI) m/z = 288.1188 [M+Na]^+^ (calculated for C_9_H_29_N_3_O_6_Na: 288.1274).

#### G1-allyl

NaH (6.0 eq, 1.10 g, 45.2 mmol) was dissolved under dry conditions and ice cooling in DMF (50 mL). G1-N3 (1.0 eq, 2.05 g, 7.51 mmol) was slowly added to the reaction mixture. After 20 min of stirring, 3-brompropene (6.0 eq, 5.50 g, 45.2 mmol) was added dropwise. The reaction was carried out overnight at 60 °C. The progress of the reaction was monitored by TLC. After completion of the reaction, the solvent was removed on the rotavapor, and the crude was extracted using DCM/H_2_O. The combined organic layers were dried over Na_2_SO_4_ and purified by column chromatography using hexane/EtOAc. The product (2.80 g) was obtained with a yield of 85 %.

**^1^H NMR** (600 MHz, CHLOROFORM-*D*) δ 5.94 – 5.86 (m, 4H), 5.29 – 5.24 (m, 4H), 5.18 – 5.14 (m, 4H), 4.15 – 4.12 (m, 4H), 4.01 – 3.98 (m, 4H), 3.70 – 3.49 (m, 15H) ppm.

**^13^C NMR** (151 MHz, CHLOROFORM-*D*) δ 135.37, 135.21, 134.80, 117.09, 117.04, 77.08, 72.47, 71.80, 71.52, 71.30, 69.97, 60.72 ppm.

**MS (ESI)** m/z = 448.2402; [M+Na]^+^ (calculated for C_21_H_35_N_3_NaO_6_: 448.2526).

#### C12-4allyl

G1-allyl (1.0 eq, 1.40 g, 3.30 mmol) and C12-alkyne (1.2 eq, 0.890 g, 4.00 mmol) were dissolved in THF (10 mL) stirred well. CuSO_4_ (0.2 eq, 0.110 g, 0.71 mmol) and Sodium ascorbate (0.4 eq, 0.260 g, 1.30 mmol) were separately dissolved in water (3 mL) and mixed well. The mixture was added to the reaction flask. The reaction was stirred at 50 °C overnight. The reaction was monitored using TLC. After completion of the reaction, the solvents were removed under reduced pressure and the residue was extracted using DCM/H_2_O. The combined organic layers were dried over Na_2_SO_4_ and purified by column chromatography using hexane/EtOAc. The product (2.00 g) was obtained with a yield of 92 % as a yellowish oil.

**^1^H NMR** (600 MHz, CDCl3) δ 7.71 (s, 1H), 5.88 -5.83 (m, 4H), 5.26 – 5.13 (m, 8H), 4.58 (s, 2H), 4.08 – 3.88 (m, 12H), 3.60 – 3.42 (m, 13H), 1.58 – 1.56 (m, 2H), 1.33 – 1.18 (m, 18H), 0.86 (t, *J* = 7.0 Hz, 3H) ppm.

**^13^C NMR** (151 MHz, CDCl3) δ 145.07, 135.06, 134.68, 122.75, 117.31, 117.14, 117.07, 77.92, 76.91, 72.44, 71.70, 71.39, 71.02, 70.27, 69.75, 64.45, 62.92, 60.58, 32.00, 29.76, 29.72, 29.69, 29.61, 29.44, 26.22, 22.77, 14.21 ppm.

**MS (ESI)** m/z = 672.4514; [M+Na]^+^ (calculated for C_36_H_63_N_3_NaO_7_: 672.4666).

#### C12-G1

C12-4allyl (1.0 eq, 0.50 g, 0.80 mmol) was dissolved in MeOH (20 mL). 2‑Dimethylamino-ethanthiol-hydrochlorid (6.0 eq, 0.780 g, 4.60 mmol) and DMPA (0.4 eq, 80.0 mg, 0.300 mmol) were added to the reaction mixture. The reaction was carried out under UV-light (365 nm) for 6 h. The progress of the reaction was monitored by TLC. After completion of the reaction, the solvent was removed, and the crude was dissolved in water and dialyzed (100-500 D) against water for 2 days (changing the water thrice a day). The residue was lyophilized, and the product (0.87 g) was obtained in a yield of 85 % as an orange oil.

**^1^H NMR** (700 MHz, MeOD) δ 8.09 (s, 1H), 4.59 (s, 2H), 4.12 – 3.85 (m, 4H), 3.73 – 3.15 (m, 42H), 2.92 (m, 7H), 2.78 – 2.49 (m, 6H), 2.09 – 1.70 (m, 6H), 1.59 (q, *J* = 6.9 Hz, 2H), 1.43 ‑ 1.14 (m, 37H), 0.90 (t, *J* = 7.0 Hz, 3H) ppm.

**^13^C NMR** (176 MHz, MeOD) δ 145.85, 124.91, 79.12, 79.08, 72.30, 72.19, 71.82, 71.49, 71.41, 70.64, 69.40, 64.78, 62.57, 52.61, 48.59, 46.94, 45.79, 33.06, 30.98, 30.79, 30.76, 30.63, 30.47, 29.44, 29.35, 27.29, 26.45, 26.40, 23.72, 14.45, 9.32, 9.20 ppm.

**MS (ESI)** m/z = 1182.8440; [M+H]^+^ (calculated for C_60_H_124_N_7_O_7_S_4_: 1182.3481).

#### C18-4allyl

G1-allyl (1.0 eq, 1.40 g, 3.30 mmol) and C18-alkyne (1.2 eq, 1.20 g, 4.00 mmol) were dissolved in THF (10 mL) stirred well. CuSO_4_ (0.2 eq, 105 mg, 0.70 mmol) and sodium ascorbate (0.4 eq, 261 mg, 1.30 mmol) were separately dissolved in water (3 mL) and mixed. The mixture was added to the reaction flask. The reaction was stirred at 50 °C overnight. The reaction was monitored using TLC. After completion of the reaction, the solvents were removed under reduced pressure and the residue was extracted using DCM/H_2_O. The combined organic layers were dried over Na_2_SO_4_ and purified by column chromatography using hexane/EtOAc. The product (2.20 g) was obtained in a yield of 90 % as a yellowish oil.

**^1^H NMR** (400 MHz, CHLOROFORM-*D*) δ 7.72 (s, 1H), 5.91 – 5.82 (m, 4H), 5.27 – 5.13 (m, 8H), 4.90 (p, *J* = 5.7 Hz, 1H), 4.59 (s, 2H), 4.17 – 3.85 (m, 13H), 3.65 – 3.36 (m, 13H), 1.62 – 1.51 (m, 2H), 1.24 (s, 28H), 0.95 – 0.76 (m, 3H) ppm.

**^13^C NMR** (101 MHz, CHLOROFORM-*D*) δ 145.03, 135.05, 134.67, 122.80, 117.19, 117.12, 76.93, 72.46, 71.72, 71.41, 71.07, 70.26, 69.75, 64.43, 60.64, 32.04, 29.81, 29.65, 29.48, 26.26, 22.81, 14.25 ppm.

**MS (ESI)** m/z = 756.5469; [M+Na]^+^ (calculated for C_42_H_75_N_3_NaO_7_: 756.5605).

#### C18-G1

C18-4allyl (1.0 eq, 500 mg, 0.70 mmol) was dissolved in MeOH (20 mL). 2‑Dimethylamino-ethanthiol-hydrochlorid (6.0 eq, 694 mg, 4.10 mmol) and DMPA (0.4 eq, 70 mg, 0.30 mmol) were added to the reaction mixture. The reaction was carried out under UV-light (365 nm) for 6 h. The progress of the reaction was monitored by TLC. After completion of the reaction, the solvent was removed, and the crude was dissolved in water and dialyzed (100-500 D) against water for 2 days (changing the water thrice a day). The residue was lyophilized, and the product (790 mg) was obtained in a yield of 82 % as an orange oil.

**^1^H NMR** (700 MHz, MeOD) δ 8.08 (s, 1H), 4.59 (s, 2H), 4.05 – 3.87 (m, 4H), 3.74 – 3.18 (m, 51H), 3.09 – 2.85 (m, 8H), 2.6 – 2.65 (m, 6H), 2.08 – 1.94 (m, 2H), 1.88 – 1.77 (m, 5H), 1.63 – 1.56 (m, 2H), 1.42 – 1.23 (m, 55H), 0.90 (t, *J* = 7.0 Hz, 3H) ppm.

**^13^C NMR** (176 MHz, MeOD) δ 145.87, 124.92, 79.12, 72.32, 72.20, 71.85, 71.50, 71.43, 70.65, 69.41, 64.79, 62.60, 52.61, 50.54, 48.60, 46.96, 45.77, 33.06, 30.99, 30.79, 30.64, 30.46, 29.45, 29.35, 27.31, 26.46, 26.42, 24.56, 24.19, 23.73, 14.44, 9.31, 9.20 ppm.

**MS (ESI)** m/z =1267.0500; [M+H]^+^ (calculated for C_66_H_136_N_7_O_7_S_4_: 1266.9379).

#### pG2-OH

Synthesized according to literature protocol.^[^[^4^](#_ENREF_4)^]^

**^1^H NMR** (600 MHz, MeOD) δ 4.27 – 4.23 (m, 4H), 4.06 – 4.04 (m, 4H), 3.84 – 3.80 (m, 1H), 3.74 – 3.72 (m, 4H), 3.68 – 3.64 (m, 4H), 3.62 – 3.50 (m, 18H), 1.39 (s, 12H), 1.33 (s, 12H) ppm.

**^13^C NMR** (151 MHz, METHANOL-D4) δ 110.52, 79.84, 76.15, 73.45, 72.80, 72.48, 72.43, 72.36, 67.60, 27.11, 25.68 ppm.

#### pG2-N3

pG2-OH (1.0 eq, 3.40 g, 4.90 mmol) was dissolved in DCM (50 mL). Under cooling with an ice bath, NEt_3_ (3.5 eq, 1.70 g, 17.1 mmol) was added slowly. After 1 h of stirring MsCl (1.5 eq, 0.810 g, 7.30 mmol) was added portion wise. The reaction was stirred over night at rt. The progress of the reaction was monitored by the thin layer chromatography using hexane/EtOAc. The crude product was extracted with DCM/H_2_O. The combined organic layers were dried over Na_2_SO_4_. The obtained pG2-OMs (1.0 eq, 3.50 g, 4.90 mmol) was dissolved in dry DMF (35 mL). NaN_3_ (3.0 eq, 2.60 g, 39.9 mmol) was added, the reaction mixture was heated up to 80 °C and stirred overnight. The progress of the reaction was monitored by thin layer chromatography using DCM/MeOH. The crude product was again extracted with DCM/H_2_O. The combined organic layers were dried with Na_2_SO_4_ and purified with column chromatography using DCM/MeOH as eluent. The product pG2-N_3_ (3.50 g, 75%) was obtained.

**^1^H NMR** (500 MHz, MeOD) δ 4.33 – 4.21 (m, 4H), 4.15 – 3.99 (m, 5H), 3.80 – 3.51 (m, 26H), 1.39 (d, *J* = 0.8 Hz, 12H), 1.33 (s, 12H) ppm.

**MS (ESI)** m/z = 744.3910; [M+Na]^+^ (calculated for C_33_H_59_N_3_NaO_14_: 744.3997).

#### G2-allyl

NaH (12 eq, 1.30 g, 55.6 mmol) was dissolved under dry conditions and ice cooling in DMF (50 mL). G2-N3 (1.0 eq, 2.60 g, 4.60 mmol) was slowly added to the reaction mixture. After 20 min of stirring, 3-brompropene (12 eq, 6.70 g, 55.6 mmol) was added dropwise. The reaction was carried out overnight at 60 °C. The progress of the reaction was monitored by TLC. After completion of the reaction, the solvent was removed on the rotavapor, and the crude was extracted using DCM/H_2_O. The combined organic layers were dried over Na_2_SO_4_ and purified by column chromatography using hexane/EtOAc. The product (3.30 g) was obtained with a yield of 82 %.

**^1^H NMR** (400 MHz, CHLOROFORM-*D*) δ 5.94 – 5.84 (m, 8H), 5.29 – 5.14 (m, 16H), 4.14 – 3.99 (m, 17H), 3.72 – 3.47 (m, 34H) ppm.

**^13^C NMR** (101 MHz, CHLOROFORM-*D*) δ 135.30, 134.87, 116.98, 78.89, 72.48, 71.82, 71.48, 70.59, 70.29, 61.35 ppm.

**MS (ESI)** m/z = 904.5132; [M+Na]^+^ (calculated for C_45_H_75_N_3_NaO_14_: 904.5249).

#### C12-8allyl

G2-allyl (1.0 eq, 1.50 g, 1.7 mmol) and C12-alkyne (1.2 eq, 0.46 g, 2.00 mmol) were dissolved in THF (10 mL) and stirred well. CuSO_4_ (0.2 eq, 55.0 mg, 0.30 mmol) and sodium ascorbate (0.4 eq, 135 mg, 0.70 mmol) were separately dissolved in water (3 mL) and mixed well. The mixture was added to the reaction flask. The reaction was stirred at 50 °C overnight. The reaction was monitored using TLC. After completion of the reaction, the solvents were removed under reduced pressure and the residue was extracted using DCM/H_2_O. The combined organic layers were dried over Na_2_SO_4_ and purified by column chromatography using hexane/EtOAc. The product (1.70 g) was obtained in a yield of 76 % as a yellowish oil.

**^1^H NMR** (400 MHz, CDCl3) δ 7.94 – 7.81 (m, 1H), 5.74 – 5.65 (m, 8H), 5.09 – 5.02 (m, 8H), 4.96 – 4.85 (m, 8H), 4.36 (s, 2H), 4.00 – 3.66 (m, 24H), 3.36 - 3.21 (m, 31H), 1.38 (q, *J* = 7.0 Hz, 2H), 1.08 (s, 28H), 0.69 (t, *J* = 6.7 Hz, 3H) ppm.

**^13^C NMR** (101 MHz, CHLOROFORM-*D*) δ 136.57, 136.13, 117.17, 117.04, 73.28, 72.50, 72.21, 71.09, 49.64, 49.42, 49.21, 49.00, 48.78, 48.57, 48.36, 30.80 ppm.

**MS (ESI)** m/z = 1128.7268; [M+Na]^+^ (calculated for C_60_H_103_N_3_NaO_15_: 1128.7389).

#### C12-G2

C12-8allyl (1.0 eq, 500 mg, 0.50 mmol) was dissolved in MeOH (20 mL). 2‑Dimethylamino-ethanthiol-hydrochlorid (12.0 eq, 1.40 g, 5.40 mmol) and DMPA (0.4 eq, 31.0 mg, 0.20 mmol) were added to the reaction mixture. The reaction was carried out under UV-light (365 nm) for 6 h. The progress of the reaction was monitored by TLC. After completion of the reaction, the solvent was removed, and the crude was dissolved in water and dialyzed (100-500 D) against water for 2 days (changing the water thrice a day). The residue was lyophilized, and the product (912 mg) was obtained in a yield of 68 % as an orange oil.

**^1^H NMR** (600 MHz, MeOD) δ 7.44 (s, 1H), 5.50 (s, 2H), 5.11 (s, 1H)z, 4.36 (t, *J* = 6.7 Hz, 2H), 4.14 - 3.96 (s, 4H), 3.71 – 3.46 (m, 43H), 3.37- 3.35 (s, 38H), 3.30 – 3.26 (m, 20H), 3.00 ‑2.89 (m, 14H), 2.78 – 2.66 (m, 14H), 1.97 – 1.76 (m, 16H), 1.47 – 1.45 (m, 2H), 1.39 – 1.28 (m, 64H), 0.91 (t, *J* = 7.0 Hz, 3H) ppm.

**^13^C NMR** (151 MHz, MeOD) δ 79.27, 72.51, 71.90, 70.71, 69.49, 52.68, 49.85, 31.08, 30.80, 30.51, 29.45, 26.42, 26.36, 23.76, 14.50, 9.24 ppm.

**MS (ESI)** m/z = 2171.4817; [M+H]^+^ (calculated for C_108_H_224_N_11_O_15_S_8_: 2171.5273).

#### C18-8allyl

G2-allyl (1.0 eq, 1.50 g, 3.30 mmol) and C18-O-ine (1.2 eq, 0.630 g, 2.00 mmol) were dissolved in THF (10 mL) stirred well. CuSO_4_ (0.2 eq, 54.0 mg, 0.300 mmol) and Sodium ascorbate (0.4 eq, 135 mg, 0.70 mmol) were separately dissolved in water (3 mL) and mixed well. The mixture was added to the reaction flask. The reaction was stirred at 50 °C overnight. The reaction was monitored using TLC. After completion of the reaction, the solvents were removed under reduced pressure and the residue was extracted using DCM/H_2_O. The combined organic layers were dried over Na_2_SO_4_ and purified by column chromatography using Hexane/EtOAc. The product (1.80 g) was obtained in a yield of 71 % as a yellowish oil.

**^1^H NMR** (400 MHz, CHLOROFORM-*D*) δ 7.81 (s, 1H), 5.74 – 5.64 (m, 8H), 5.09 – 5.04 (m, 8H), 4.96 – 4.91 (mf, 8H), 4.36 (s, 2H), 4.00 – 3.66 (m, 24H), 3.41 - 3.29 (m, 31H), 1.37 – 1.35 (m, 2H), 1.16 – 1.04 (m, 28H), 0.69 (t, *J* = 6.7 Hz, 3H) ppm.

**^13^C NMR** (101 MHz, CHLOROFORM-*D*) δ 136.57, 136.13, 117.17, 117.04, 73.28, 72.50, 72.21, 71.09, 49.64, 49.42, 49.21, 49.00, 48.78, 48.57, 48.36, 30.80 ppm.

**MS (ESI)** m/z = 1212.8176; [M+Na]^+^ (calculated for C_66_H_115_N_3_NaO_15_: 1212.8328).

#### C18-G2

C18-8allyl (1.0 eq, 0.50 g, 0.40 mmol) was dissolved in MeOH (20 mL). 2‑Dimethylamino-ethanthiol-hydrochlorid (12.0 eq, 1.30 g, 5.00 mmol) and DMPA (0.4 eq, 28.0 mg, 0.200 mmol) were added to the reaction mixture. The reaction was carried out under UV-light (365 nm) for 6 h. The progress of the reaction was monitored by TLC. After completion of the reaction, the solvent was removed, and the crude was dissolved in water and dialyzed (100-500 D) against water for 2 days (changing the water thrice a day). The residue was lyophilized, and the product (0.87 g) was obtained in a yield of 74 % as an orange oil.

**^1^H NMR** (600 MHz, MeOD) δ 8.13 (s, 1H), 4.61 – 4.60 (m, 2H), 4.10 (s, 2H), 4.10 – 3.95 (m, 1H), 3.74 – 3.33 (m, 92H), 3.29 – 3.22 (m, 16H), 3.11 – 2.90 (m, 14H), 2.72 -2.70 (m, 12H), 2.23 – 1.72 (m, 16H), 1.61 (d, *J* = 7.2 Hz, 2H), 1.38 – 1.28 (m, 75H), 0.91 (t, *J* = 6.9 Hz, 3H) ppm.

**^13^C NMR** (151 MHz, MeOD) δ 79.29, 72.52, 71.95, 70.73, 69.52, 52.67, 49.85, 46.94, 33.09, 31.09, 30.84, 30.49, 29.46, 26.43, 26.38, 23.74, 14.47, 9.24 ppm.

**MS (ESI)** m/z = 2256.5760; [M+H]^+^ (calculated for C_114_H_236_N_11_O_15_S_8_: 2256.5730).

# Physicochemical Characterization


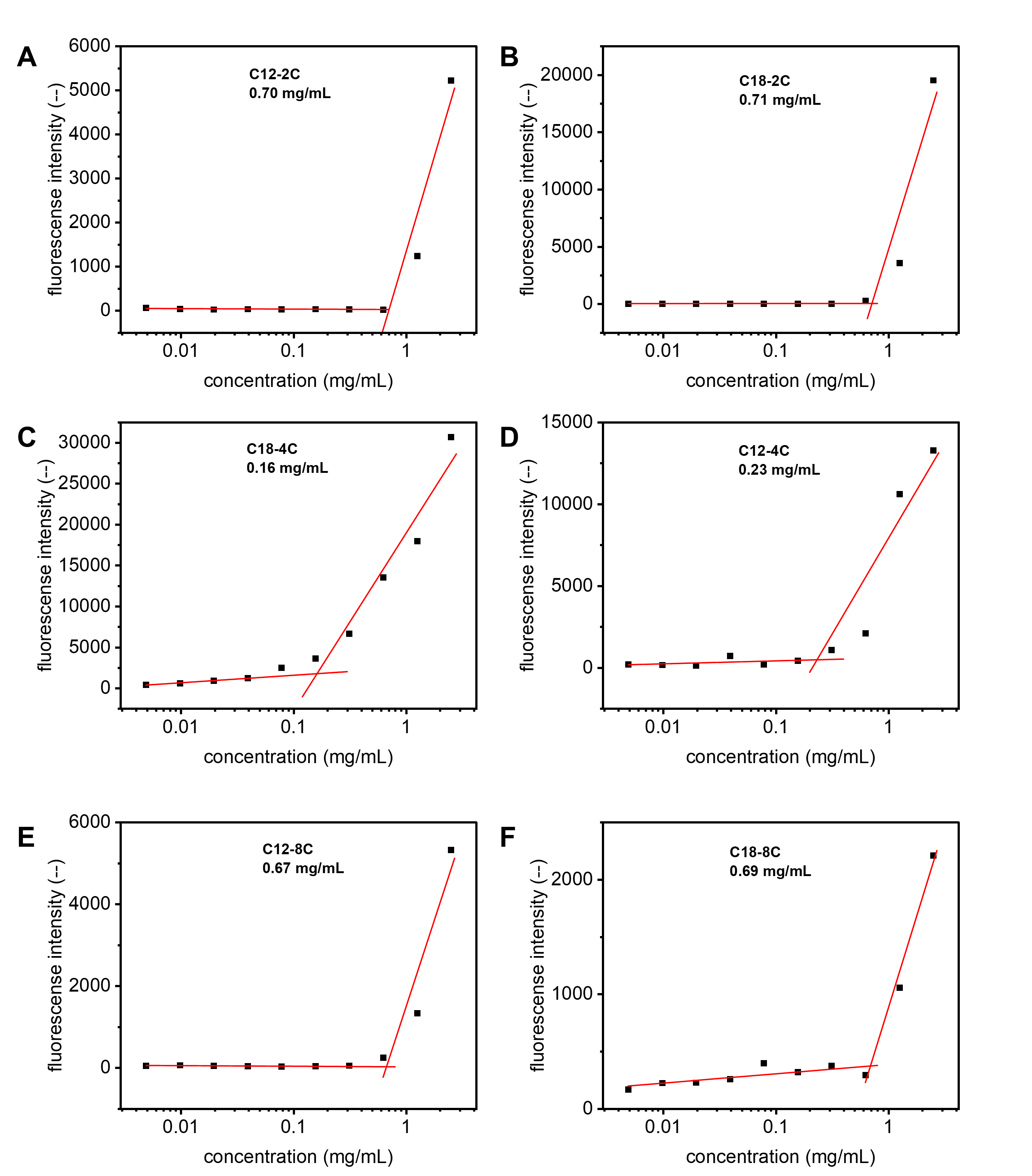


**Figure S10.** Critical micelle concentration of the different cationic surfactants using Nile red encapsulation method and fluorescence measurements. (A) C12-2C: 0.70 mg/mL; (B) C18‑2C: 0.71 mg/mL; (C) C12-4C: 0.16 mg/mL; (D) C18-4C: 0.23 mg/mL; (E) C12-8C: 0.67 mg/mL; (F) C18-8C: 0.69 mg/mL.


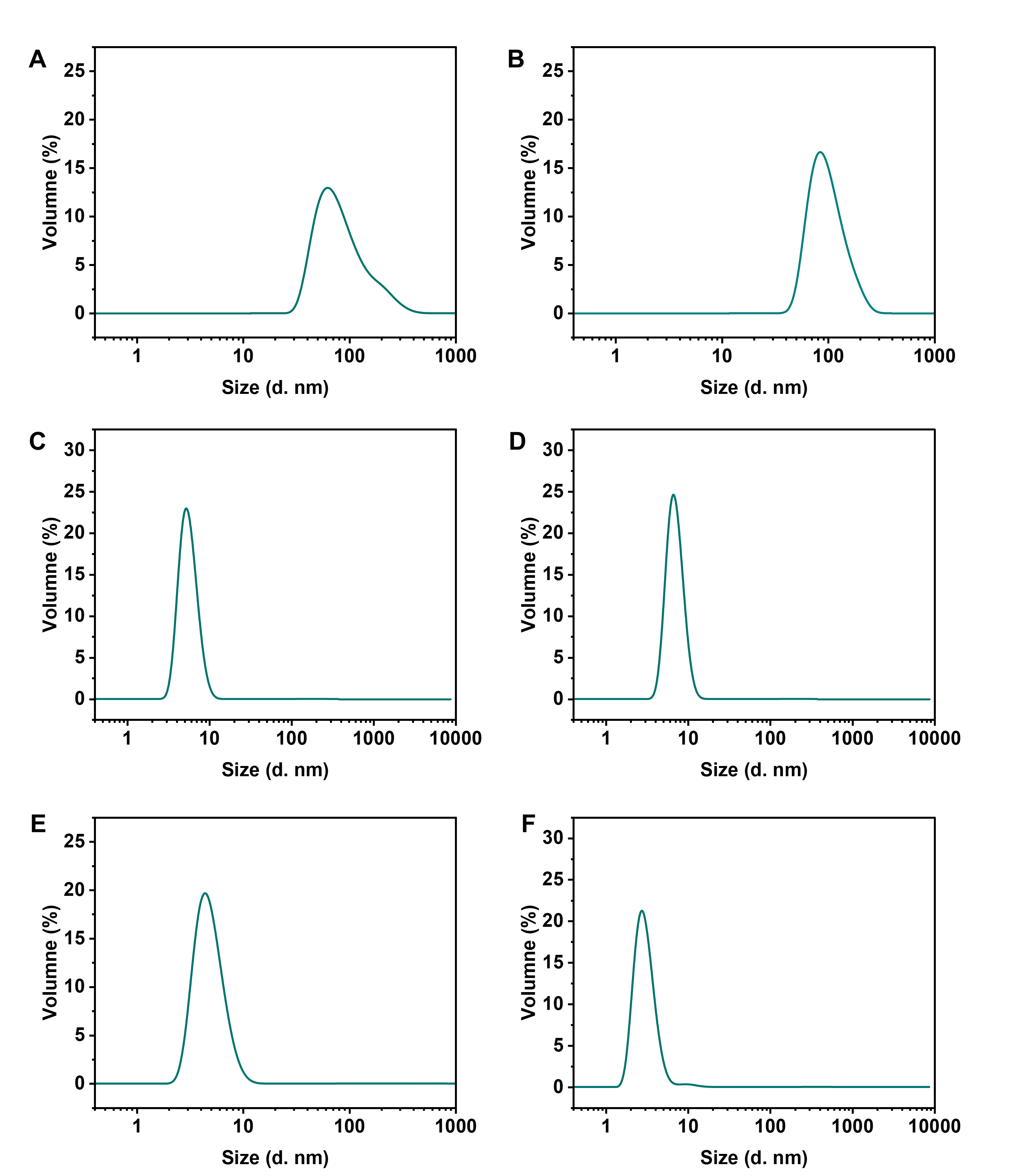


**Figure S11.** Dynamic light scattering (DLS) of the cationic surfactants in PBS at a concentration of 1 mg/ml visualizing the formation of self-assembled aggregates. For the 2C (G0) surfactants (C12-2C and C18-2C) hydrodynamic diameters of about 100 nm have been found, while in case of more charged groups in the hydrophilic head group of the surfactants (C12/C18-4C and C12/C18-8C) the hydrodynamic diameter is much smaller, around 5-7 nm.

**Table S1.** Overview of physicochemical properties of the cationic surfactants. Dynamic light scattering results of the volume based hydrodynamic diameter at a surfactant concentration of 1 mg/mL in PBS. Critical micelle concentration estimated using Nile red encapsulation method. Zetapotential of the surfactants at a concentration of 1 mg/mL in diluted PBS (10 mM).

| **Surfactant** | **Size [d. nm]** | **CMC [mg/mL]** | **Zeta potential ± SD [mv]** |
| --- | --- | --- | --- |
| C12-2C | 63 | 0.70 | 32 ± 2 |
| C18-2C | 83 | 0.71 | 39 ± 3 |
| C12-4C | 5 | 0.16 | 52 ± 2 |
| C18-4C | 7 | 0.23 | 61 ± 6 |
| C12-8C | 5 | 0.67 | / |
| C18-8C | 5 | 0.69 | 58 ± 3 |

# Appendix

## NMR

**Figure S12**. ^1^H NMR of C12-alkyne.

**Figure S13.** ^13^C NMR of C12-alkyne.

 **Figure S14.**^1^H NMR of C18-alkyne.

**Figure S15.** ^13^C NMR of C18-alkyne.

**
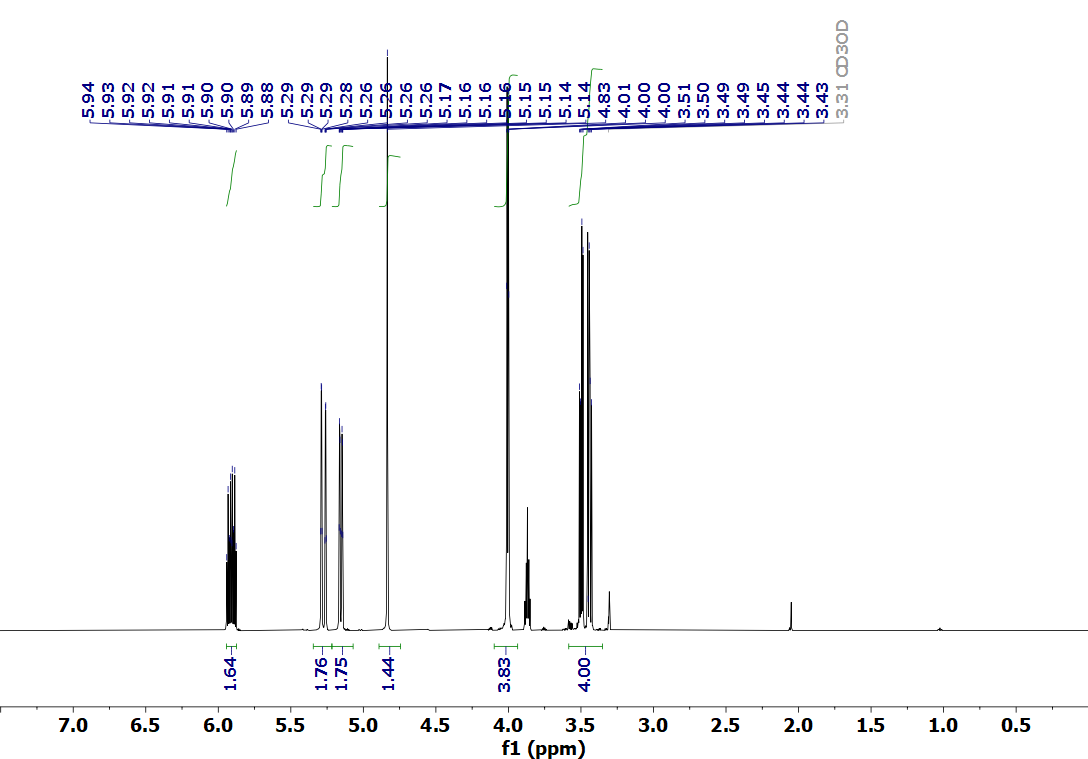
**

**Figure S16.** ^1^H NMR spectra of G0-OH.


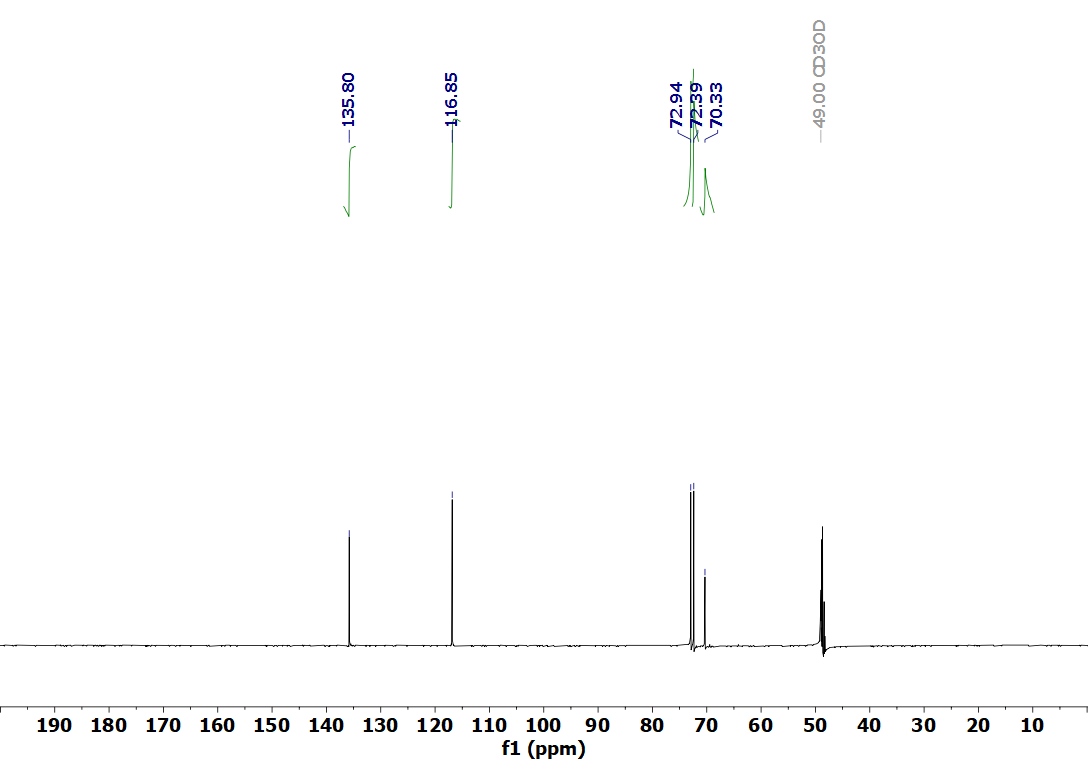


**Figure S17.** ^13^C NMR spectra of G0-OH.

***
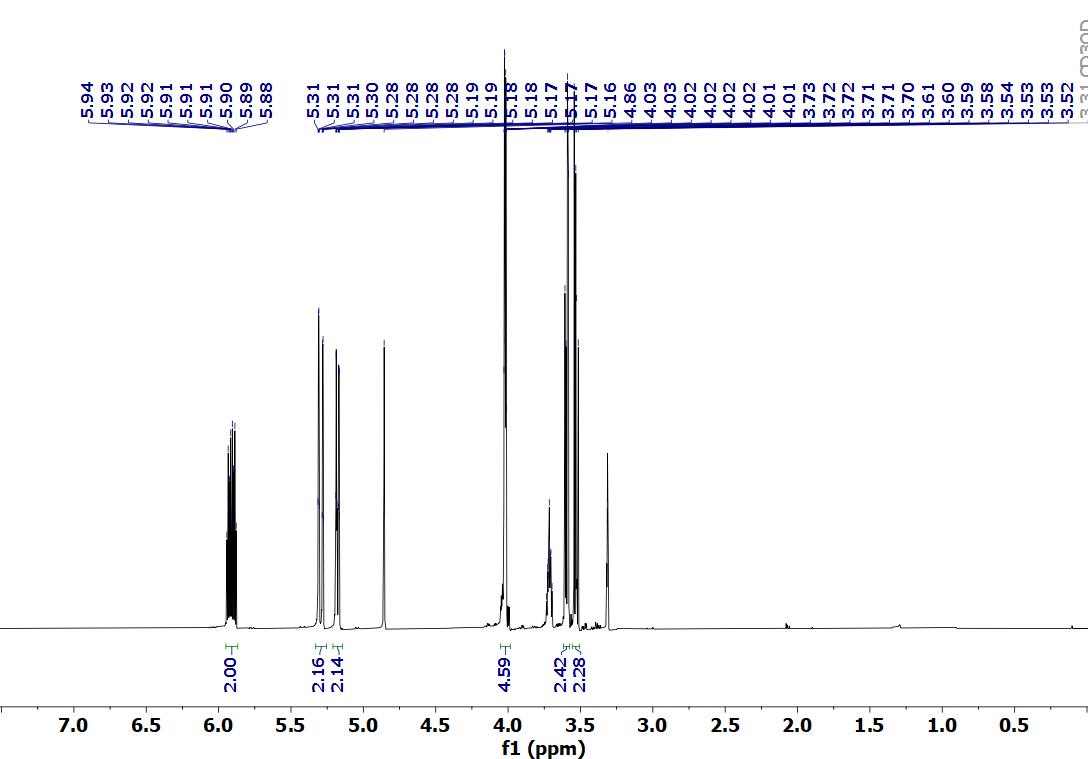
***

**Figure S18.** ^1^H NMR spectra of G0-N_3._


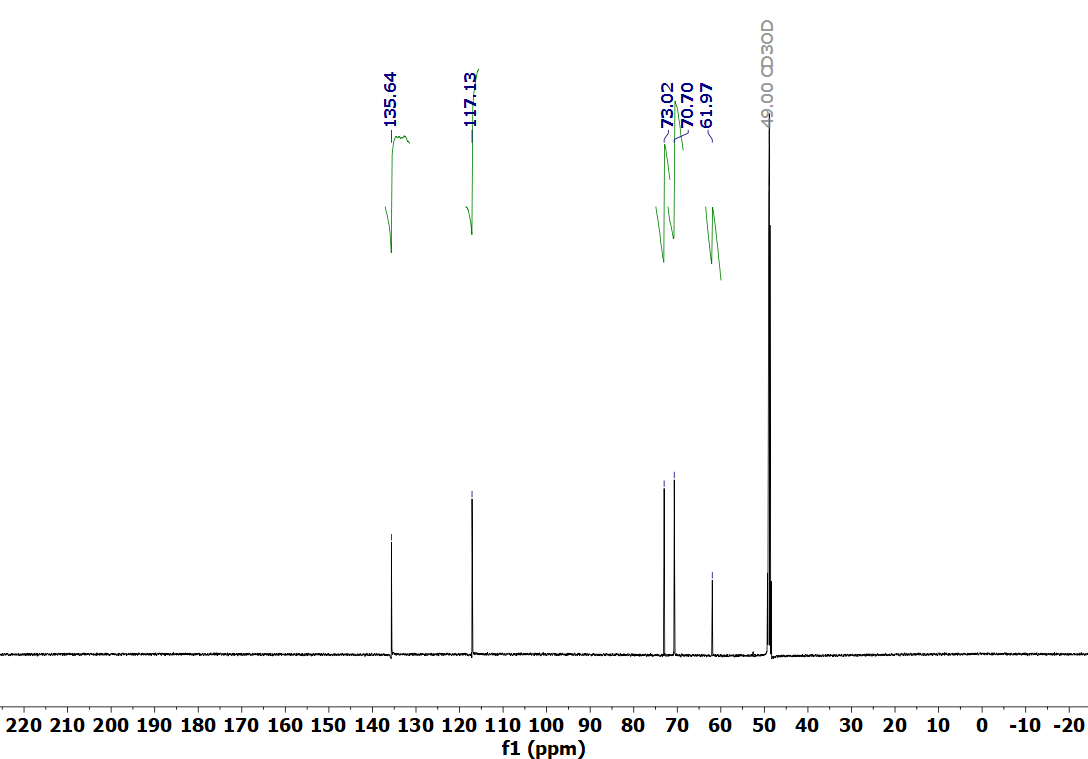


**Figure S19.** ^13^C NMR spectra of G0-N_3_.

***
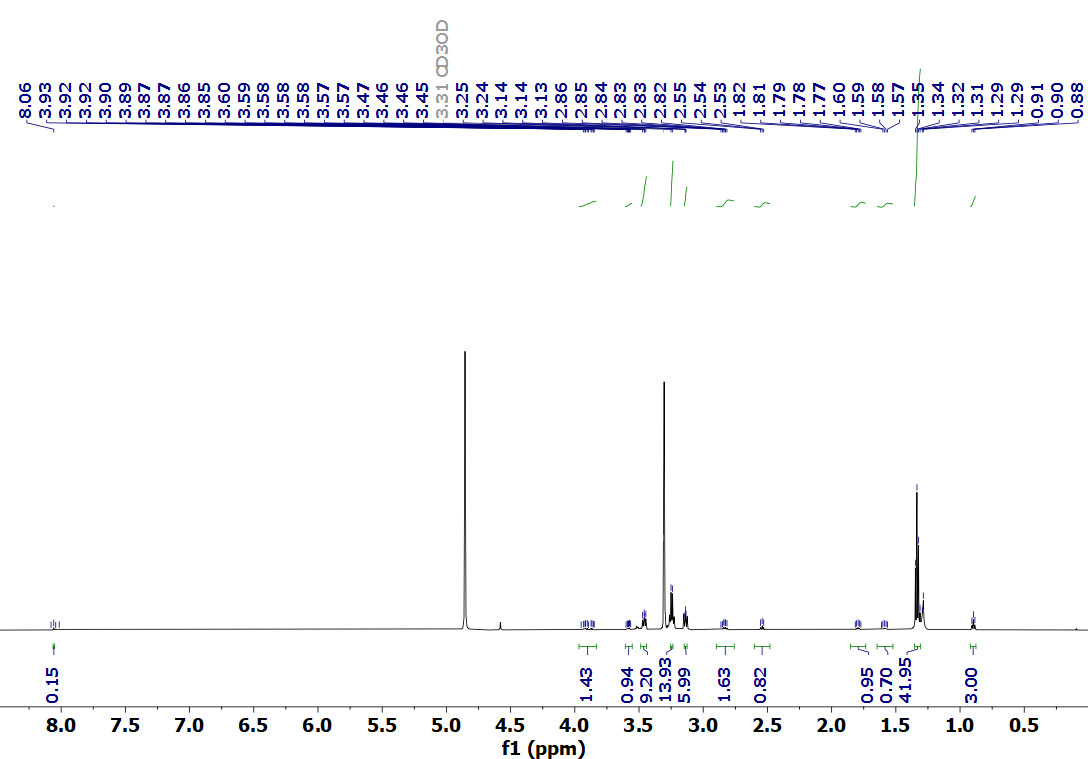
***

**Figure S20.** ^1^H NMR spectra of C18-G0.


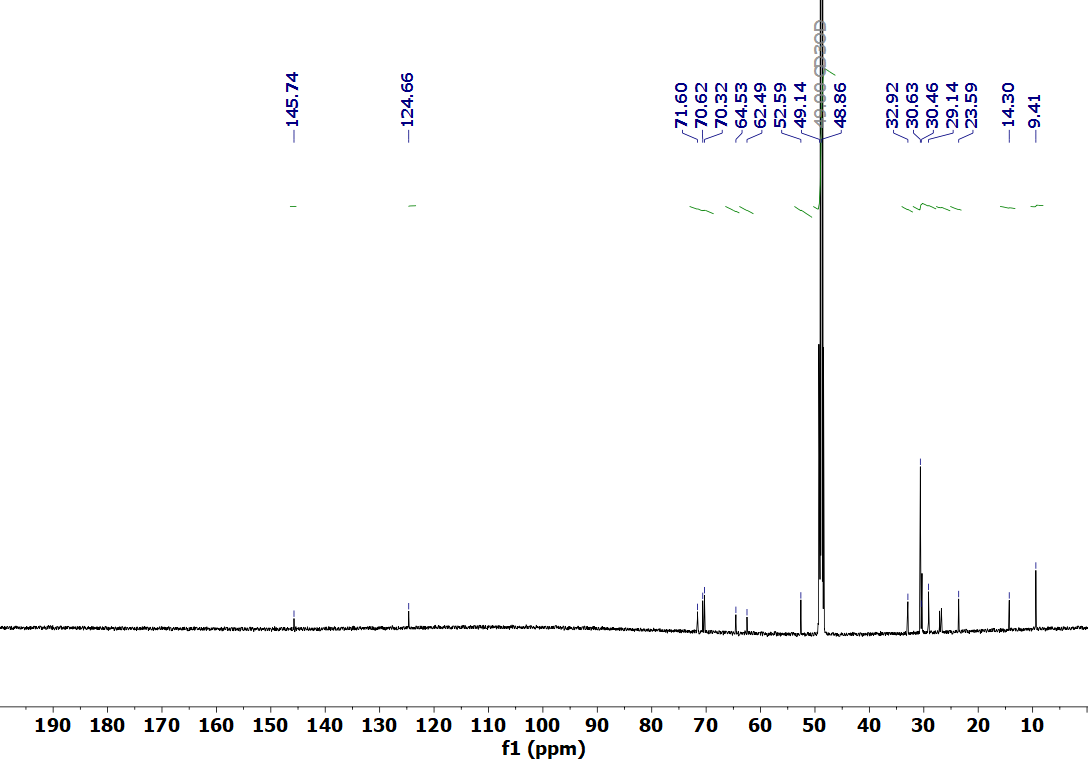


**Figure S21.** ^13^C‑NMR spectra of C18-G0.

**
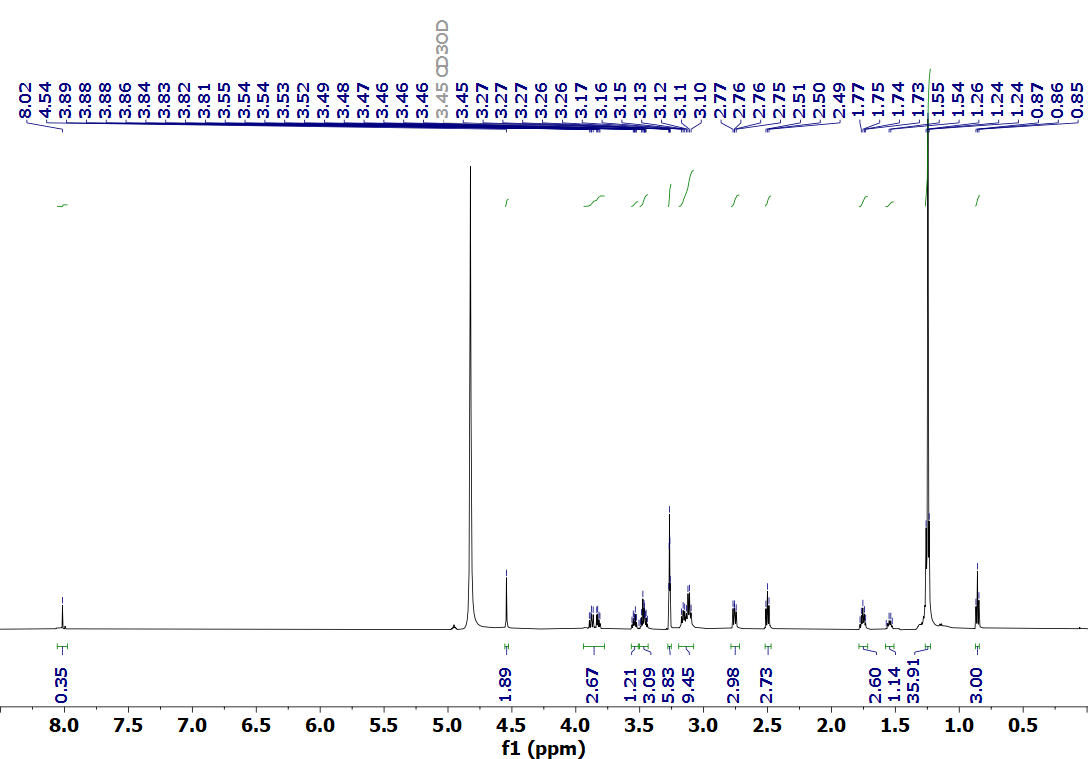
**

**Figure S22.** ^1^H NMR spectra of C12-G0.


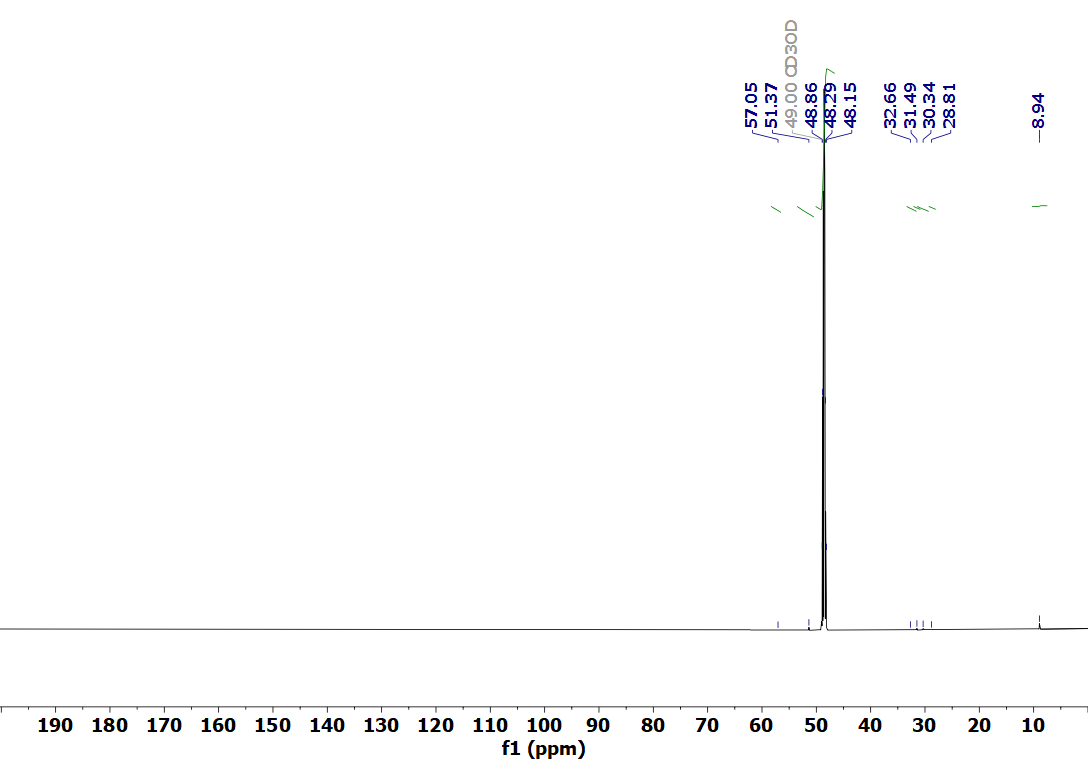


**Figure S23.** ^13^C NMR spectra of C12-G0.

**Figure S24.** ^1^H NMR of pG1-OH.

**Figure S25.** ^13^C NMR of pG1-OH.

**Figure S26.** ^1^H NMR of pG1-N3.

**Figure S27.** ^13^C NMR of pG1-N3.

**Figure S28.** ^1^H NMR of G1-N3.

**Figure S29.** ^13^C NMR of G1-N3.

**Figure S30.** ^1^H NMR of G1-allyl.

**Figure S31.** ^13^C NMR of G1-allyl.

**Figure S32.** ^1^H NMR of C12-4allyl.

**Figure S33.** ^13^C NMR of C12-4allyl.

**Figure S34.** ^1^H NMR of C12-4C.

**Figure S35.** ^13^C NMR of C12-4C.

**Figure S36.** ^1^H NMR of C18-4allyl.

**Figure S37.** ^13^C NMR of C18-4allyl.

**Figure S38.** ^1^H NMR of C18-4C.

**Figure S39.** ^13^C NMR of C18-4C.

**Figure S40.** ^1^H NMR of pG2-OH.

**Figure S41.** ^13^C NMR of pG2-OH.

**Figure S42.** ^1^H NMR of pG2-N3.

**Figure S43.** ^1^H NMR of G2-allyl.

**Figure S44.** ^13^C NMR of G2-allyl.

**Figure S45.** ^1^H NMR of C12-8allyl.

**Figure S46.** ^13^C NMR of C12-8allyl.

**Figure S47.** ^1^H NMR of C12-8C.

**Figure S48.** ^13^C NMR of C12-8C.

**Figure S49.** ^1^H NMR of C18-8allyl.

**Figure S50.** ^13^C NMR of C18-8allyl.

**Figure S51.** ^1^H NMR of C18-8C.

**Figure S52.** ^13^C NMR of C18-8C.

**Literature**

[1] C. N. Baker, F. C. Tenover, *J Clin Microbiol* **1996**, *34*, 2654-2659.

[2] K. el Battioui, S. Chakraborty, A. Wacha, D. Molnár, M. Quemé-Peña, I. C. Szigyártó, C. L. Szabó, A. Bodor, K. Horváti, G. Gyulai, S. Bősze, J. Mihály, B. Jezsó, L. Románszki, J. Tóth, Z. Varga, I. Mándity, T. Juhász, T. Beke-Somfai, *Nature Communications* **2024**, *15*, 3424.

[3] M. Wyszogrodzka, R. Haag, *Chem-Eur J* **2008**, *14*, 9202-9214.

[4] A. Tschiche, A. M. Staedtler, S. Malhotra, H. Bauer, C. Bottcher, S. Sharbati, M. Calderon, M. Koch, T. M. Zollner, A. Barnard, D. K. Smith, R. Einspanier, N. Schmidt, R. Haag, *J Mater Chem B* **2014**, *2*, 2153-2167.
